# Biomarkers of Internet Gaming Disorder—A Narrative Review

**DOI:** 10.3390/jcm13175110

**Published:** 2024-08-28

**Authors:** Katarzyna Skok, Napoleon Waszkiewicz

**Affiliations:** 1 Faculty of Education, University of Bialystok, ul. Świerkowa 20, 15-328 Bialystok, Poland; 2 Department of Psychiatry, Medical University of Bialystok, pl. Wołodyjowskiego 2, 15-272 Bialystok, Poland; napwas@wp.pl

**Keywords:** video games, Internet gaming disorder, IGD, addiction, gaming, biomarkers

## Abstract

Since game mechanics and their visual aspects have become more and more addictive, there is concern about the growing prevalence of Internet gaming disorder (IGD). In the current narrative review, we searched PubMed and Google Scholar databases for the keywords “igd biomarker gaming” and terms related to biomarker modalities. The biomarkers we found are grouped into several categories based on a measurement method and are discussed in the light of theoretical addiction models (tripartite neurocognitive model, I-PACE). Both theories point to gaming-related problems with salience and inhibition. The first dysfunction makes an individual more susceptible to game stimuli (raised reward seeking), and the second negatively impacts resistance to these stimuli (decreased cognitive control). The IGD patients’ hypersensitivity to reward manifests mostly in ventral striatum (VS) measurements. However, there is also empirical support for a ventral-to-dorsal striatal shift and transition from goal-directed to habitual behaviors. The deficits in executive control are demonstrated in parameters related to the prefrontal cortex (PFC), especially the dorsolateral prefrontal cortex (DLPFC). In general, the connection of PFC with reward under cortex nuclei seems to be dysregulated. Other biomarkers include reduced P3 amplitudes, high-frequency heart rate variability (HRV), and the number of eye blinks and saccadic eye movements during the non-resting state. A few studies propose a diagnostic (multimodal) model of IGD. The current review also comments on inconsistencies in findings in the nucleus accumbens (NAcc), anterior cingulate cortex (ACC), and precuneus and makes suggestions for future IGD studies.

## 1. Introduction

Internet gaming has become more and more popular in the past two decades, with 1.1 billion online gamers (mainly from China, South Korea, and Japan), and in 2023 (by the end of August), the online gaming business generated approximately USD 26.14 billion [1]. Many players have become addicted, and the prevalence of Internet gaming disorder (IGD) among adolescents and young adults has reached 9.9% [2]. The emergence of IGD is attributed to the following 12 factors: stress, long average game time, family dysfunction, poor academic performance, being bullied, bullying, interpersonal problems, hyperactivity/inattention, anxiety, depression, emotional distress, and low self-esteem [2]. The disorder was included in the *Diagnostic and Statistical Manual of Mental Disorders, 5th Edition* (DSM-5), Section III in 2013 as requiring further study [3]. The proposed nine criteria are preoccupation, withdrawal, tolerance, unsuccessful attempts to stop or reduce, loss of interest in other hobbies or activities, excessive gaming despite problems, deception, escape or relief from a negative mood, and jeopardized or lost a relationship, job or educational, or career opportunity [4]. Gaming disorder (GD; “digital-gaming” or “video-gaming”) was also listed in the 11th revision of the *International Classification of Diseases* (ICD-11) [5] and included both online and offline variants. It is characterized by impaired control over gaming, increasing priority given to gaming compared to other activities, and continuation despite negative consequences. Some studies reported that IGD is attributed to a worsening of preexisting mental health issues, while others suggest that it leads to adverse consequences for mental well-being. Either way, the problems include/are linked to depression, social cognition, and emotional regulation (in combination with ADHD), autism spectrum disorder, sleep disturbances, lower life satisfaction [6], impulsiveness, anxiety, maladaptive cognitions, and others [7].

Growing concern over IGD and a search for effective early diagnosis and treatment has caused an increasing number of studies on objective measurable biological indicators (biomarkers) associated with IGD risk factors or symptoms. It can be “any substance, structure or process that can be measured in the body or its products and influence or predict the incidence of outcome or disease” [8]. A broader definition also includes the effects of treatments and interventions [9]. The IGD biomarkers fall into the following several categories based on a measurement method: electroencephalography (EEG) (resting and non-resting state), neuroimaging (structure activation, functional connectivity, gray matter (GM) volume, brain controllability, dopamine release, glucose metabolism in resting and non-resting state), genetics, blood sampling, blood pressure/heart rate (resting and non-resting state), and somatic markers.

## 2. Methods

PubMed and Google Scholar were searched for the keywords “igd biomarker gaming”. Additionally, we searched PubMed for “Internet gaming disorder” and terms related to biomarker modalities. For PubMed, we used no publication date filters, and all article types were initially accepted. In the case of Google Scholar, we limited the search to articles published since 2021. The PubMed search identified 269 results, and the Google Scholar search identified 297 results (1 February 2024). All applicable PubMed and Google Scholar publications were included in this review. We also followed the citations, as not all relevant articles included the “biomarker” term in the keywords. Finally, a total of 169 empirical and 9 non-empirical publications matched the inclusion criteria.

## 3. Results

### 3.1. EEG

The resting state-altered brain activity of players with GD is well documented. Burleigh et al. [10] suggest that some neurophysiological differences between disordered gamers and healthy individuals may be GD trait markers. A power spectral analysis revealed that before serotonin treatment, individuals with IGD showed higher delta band power across the whole brain and higher theta activity centrally compared to healthy individuals. After the six-month selective serotonin reuptake inhibitor (SSRI) treatment (escitalopram, fluoxetine, and paroxetine), the IGD group had reduced frontal delta activity compared to baseline [11]. A study combining EEG and heart rate variability (HRV) methods during a resting state demonstrated that the IGD group exhibited higher theta band characteristic path length, as well as negative correlations between the standard deviation of the theta and delta normal-to-normal interval index and characteristic path length values [12]. Lower absolute beta power was observed in patients with IGD compared to those with alcohol use disorder (AUD) and the healthy controls [13]. Both IGD and AUD individuals had reduced mean absolute power (theta and beta bands) in the frontal and prefrontal regions [14].

Participants with both ADHD and IGD (compared to ADHD-only participants) had lower relative delta wave power and higher relative beta power in the temporal regions. Also, a coherence analysis showed that the ADHD/IGD group had higher intrahemispheric coherence values in the delta band than the ADHD-only controls and higher intrahemispheric coherence in the theta, alpha, and beta waves than the ADHD and control groups [15]. IGD individuals showed higher intrahemispheric coherence for gamma (compared to the healthy and AUD ones). Additionally, gamma coherence correlated positively with Internet addiction scores for all groups [16]. After conducting a longitudinal study on IGD-diagnosed and healthy participants, Park et al. suggested that raised intrahemispheric coherence in beta and gamma waves may be the trait biomarker of IGD; a serotonin treatment lowered IGD symptoms in IGD patients, while their EEG coherence remained unchanged [17]. Youh et al. compared individuals suffering from major depressive disorder (MDD) only and those suffering from both MDD and IGD. The first group had lower interhemispheric coherence of alpha waves between the left and right fronto-polar electrodes and higher intrahemispheric coherence of alfa (between the left parietal-occipital electrodes) and beta (between the right fronto-temporal, temporo-occipital and parieto-occipital electrodes). The authors suggest that raised intrahemispheric coherence may stem from high gaming engagement [18]. Another resting state coherence study demonstrated that IGD patients, compared to the healthy controls, had raised theta, alpha, and beta connectivity between the orbitofrontal cortex (OFC) and parietal regions, as well as increased alpha and beta connectivity between the anterior cingulate gyrus and temporal regions [19]. Higher alpha coherence in the right hemisphere was also observed in IGD individuals with low resilience compared to those with high-resilience and non-IGD participants [20]. This may suggest that higher alpha coherence in the right hemisphere as a biomarker may be specific to some IGD subgroups. Similarly, game genre and use type (single game vs. multiple games) produce different neurophysiological patterns. Higher beta activity was demonstrated in single-game players (compared to multiple-game players and the control group), first-person shooter players had increased delta power in the frontal region (compared to controls), and massively multiplayer online role-playing game and first-person shooter players had lowered intrahemispheric coherence in the left frontoparietal region [21]. A multimodal-based machine learning approach showed EEG coherence differences between IGD and AUD [22]. The IGD group had reduced delta connectivity between the right OFC and the right angular gyrus and inferior parietal lobe in the default mode network (DMN), increased beta connectivity between the right PFC and the right temporal lobe in the reward-salience network, increased delta connectivity between the right temporal lobe and the right posterior cingulate cortex and cuneus, and reduced beta connectivity between the right PFC and the right ACC [22].

Burleigh et al. [10] point to the slow-wave activity that is associated with many cognitive processes (e.g., attention or control processes). Its increase is related to impairments in attention, control processes, and inhibitory control [23]. Yet, it still remains unclear if the raised delta and theta activity stems from trait predispositions or is a result of excessive gaming. IGD individuals also have lower beta activity [14], which is associated with ADHD impairments and may indicate an impulsivity trait. Nonetheless, ADHD/GD patients had higher beta activity than IGD patients [15]. The authors suggest that this may be associated with the need for ADHD individuals to enhance their attentional ability. An interesting finding comes from another coherence study; raised intrahemispheric coherence in fast waves did not drop in IGD patients after successful SSRI treatment (escitalopram, fluoxetine, or paroxetine), which may suggest a non-state trait marker of IGD [17].

Reduced P3 amplitudes (the midline centroparietal electrode regions) in the IGD group were observed during an oddball task by Park et al. [24]. In another study on EEG during oddball tasks, both IGD and AUD patients exhibited lowered P3 amplitudes at the midline central and parietal area compared to healthy participants, but only in the IGD group did P3 correlate with a higher spatial span error rate. Also, only the IGD group had reduced N1 amplitudes at the midline central and parietal areas [25]. Decreased P3 amplitudes correlated positively with the right inferior temporal gyrus and the occipital regions and negatively with regional homogeneity values of the left hippocampus and the right amygdala during a resting-state fMRI measurement [26]. A semi-natural gaming study (measurement during playing) showed similar results; IGD individuals had decreased P3 amplitude in response to rewards, prolonged N1 latency, and increased N1 amplitude [27]. A decreased resting-state fractional amplitude of low-frequency fluctuation values was observed in the cerebellum posterior lobe, and raised ones were observed in the superior temporal gyrus. Additionally, interactions were demonstrated in the cerebellum, ACC, lingual gyrus, middle temporal gyrus, and MFG [28].

Different EEG patterns during risk-taking decisions were exhibited in IGD individuals and recreational game users (RGU). The RGU group showed larger N2 amplitudes for risk disadvantageous decisions in the loss domain than the gain domain, while there was no such difference in the control group, which may suggest reduced loss aversion [29]. As the, respectively, trait and state markers of IGD craving, raised late low potentials [30] and parieto-occipital theta power [31] were proposed. The following EEG correlates of game craving were demonstrated by Park et al. [32]: absolute powers of the central and parieto-occipital delta, theta, and beta. A literature review by Kashif et al. [33] summarized the results of EEG studies and suggested that raised slow-wave resting-state activity and decreased P3 and N1 can serve as the diagnostic IGD markers, while raised resting-state theta activity is the predictive one.

Prolonged event-related N2 latency may be a potential biomarker of impulsivity mediating IGD [34], and lowered frontal theta activity during gaming may serve to detect diminished cognitive control [35]. A study conducted on healthy and IGD individuals while playing a popular game showed that IGD symptoms can be recognized by the parameters from alpha, sensory motor rhythm, and mid-beta (Fp1, C3, C4, and O1 channels) [36]. A negative correlation between alpha coherence at the left frontocentral and bilateral centrotemporal electrodes (resting state) with P3 latency (go/no-go task) was observed in the IGD group, while healthy participants demonstrated the inverse relationship [37].

### 3.2. Neuroimaging

#### 3.2.1. Brain Connectivity and Activation—The Tripartite Model

Wei et al. [38] proposed a tripartite model that encompasses the following three neurocognitive brain systems: (1) the impulsive one (fast, habitual, unconscious reactions), (2) the reflective one (planning, inhibitory control), and the interoceptive awareness one (converting somatic signals of reward deprivation into subjective desire). The model was discussed and examined in several other studies [39]. An fMRI study [40] demonstrated that IGD score correlated positively with VS (associated with reward processing) activation in response to game cues and negatively with left frontal pole and right DLPFC (planning, decision-making) activation. Additionally, in gamers under deprivation conditions, the left insular cortex (mediator of cue-induced cravings) was the most active when they were exposed to game cues. Finally, the study showed that under deprivation, the left insula activation was positively associated with left VS activation and negatively associated with left DLPFC activation. The above observations are consistent with other studies. The IGD group (especially females) demonstrated decreased DLPFC activation in pre–post gaming tests compared to non-addicted frequent players. During the post-gaming (under deprivation) cue-elicited craving task, IGD females showed higher activation in the caudate compared to non-IGD females [41]. Decreased activation was also observed in the ACC, parahippocampal gyrus, and DLPFC [42]. The results of both above studies come from IGD recreational use groups. IGD individuals, compared to healthy ones, had greater VS activation in reaction to a significant missed chance [43]. During a risky decision-making task, the IGD group showed greater responses within the VS for potential gains and less activation in the DLPFC for potential losses [44]. A blunted reward prediction error (reinforcement learning) was observed in the right caudate, left OFC, and right DLPFC in IGD individuals. The IGD group demonstrated increased connectivity between the right caudate, right putamen, bilateral DLPFC, and right dorsal ACC. A connection between the right DLPFC and right dorsal ACC could predict the variation of reward prediction error signals in the left OFC [45]. IGD patients performed worse in the Iowa gambling task than the healthy controls. In a healthy group [46], there was a correlation between loss aversion and the edge community profile similarity of the edge between the left inferior frontal gyrus (IFG) and the right hippocampus at the right caudate (edge-centric functional connectivity). It was suppressed by response consistency in the IGD group. Reduced loss aversion correlated negatively with the promoted bottom-up neuromodulation from the right hippocampus to the left IFG (IGD participants). Decreased activation in the DLPFC and bilateral IFG was also demonstrated during a delay discounting task [47]. However, a task involving a high working memory load was associated with increased DLPFC and VS activation, which may indicate higher cognitive demands on the response inhibition system [48].

A study by Ko et al. [49] on response inhibition showed that IGD individuals had higher brain activation over the left orbital frontal lobe and bilateral caudate nucleus than healthy participants while performing go/no-go tasks. Also, the IGD group, compared to the control group, had lower activation over the right insula during error processing (both groups exhibited activation of the insula and ACC). An fMRI inhibition study showed lower activation of supplementary motor area (SMA/pre-SMA) in the IGD group, while the healthy participants exhibited higher SMA, DLPFC, and caudate activation [50]. Increased activation was observed in the right OFC, right NAcc, bilateral ACC and medial frontal cortex, right DLPFC, and right caudate nucleus in response to cue-related stimuli [51]. On the other hand, the right ACC, right precuneus, left precentral gyrus, and right postcentral gyrus (PCG) showed lower activation during the same measurement procedure [52]. Higher activation was observed in pre-SMA and the VS in the decision-making and estimation phases during the two-armed bandit task. Additionally, PFC activation was reduced during exploitative strategies in this task [53]. IGD individuals, compared to healthy controls, took more risks in a roulette task and had raised NAcc and caudate activation during the reward anticipation and outcome monitoring phases (but not during the choice evaluation) [54]. Lower activation of the anterior insular cortex and dorsal attention network was also demonstrated during risky decision-making [55]. Ma et al. [56] identified the following four large-scale functional brain networks that showed different engagement/disengagement reactions to game cues: temporo-occipital, temporo-insula (sensory processing), frontoparietal (memory and execution), and dorsal-limbic (reward and motivation). The temporo-occipital and frontoparietal networks correlated positively with IGD severity, and the temporo-insula network correlated negatively with craving. Another study demonstrated higher activation in the right ACC, PCC, PFC, middle temporal gyrus, left DLPFC, and thalamus, as well as lowered right PCC–right inferior parietal lobule (IPL) functional connectivity during a regulation of craving task [57].

IGD and healthy adolescents exhibited similar behavioral performance (visual working memory and attention), yet IGD individuals had raised global/long-range resting-state functional connectivity density in the bilateral DLPFC and the right inferior temporal cortex/fusiform, which may indicate a compensatory mechanism [58]. A later study showed that precuneus functional connectivity with caudate was higher for game stimuli than food-related stimuli in IGD players (recreational players demonstrated the opposite results) [59]. Excessive players had increased valence attribution and neural reactivity in the PCC/precuneus compared to gaming-naïve individuals. Six weeks of gaming also raised neural reactivity in the above regions in the latter group (compared to the baseline) [60]. The volume of the precuneus, its activation, and its connectivity from the hippocampal gyrus during cue-related tasks correlated positively with IGD severity. A negative correlation with connectivity from the middle frontal gyrus to the precuneus was observed [61]. Post-gaming compared to pre-gaming increased activation to game-related stimuli was observed in the PFC, striatum, and precuneus [62]. However, a resting-state study showed decreased OFC, precuneus, and DLPFC activity in persistent IGD individuals compared to naturally recovered individuals [63]. The inconsistent observations can be attributed to different measurement paradigms. The DMN plays an important role in IGD craving, and its activity/connectivity related to craving is better captured during a non-rest-state fMRI.

Lowered resting-state functional connectivity was observed between the right DLPFC and the right IFG and the right ACC and the superior parietal lobule but increased connectivity between the left dorsal putamen and the PCG. DLPFC–IFG connectivity correlated with in-game high-frequency HRV [64]. The resting-state functional connectivity of IGD subjects depended on previous ADHD history. IGD individuals with no childhood ADHD had increased functional connectivity within the DMN regions (PCC, mPFC, thalamus) compared to controls and raised functional connectivity between the PCC and salience areas (anterior insula, OFC) compared to the childhood ADHD/IGD subjects. The ADHD/IGD group demonstrated higher PCC–cerebellum functional connectivity, and it correlated positively with impulsiveness [65]. Network-based statistics and IGD test scores helped identify the IGD sub-network in addicts with comorbid ADHD. IGD severity was predicted by the edges connecting the left precentral gyrus, left PCG, bilateral superior frontal gyrus (SFG), medial orbital parts, and left fusiform to the inferior temporal gyrus. The authors concluded that the sub-network may be a phenotype of comorbid ADHD [66]. The resting-state right precentral gyrus and the left PCG were the most informative regions in the prediction of IGD severity [67]. The static and dynamic regional homogeneity calculations in a resting state showed that the IGD group had raised static and dynamic intrinsic local connectivity in bilateral medial SFG, SFG, and SMA. Increased dynamic regional homogeneity was demonstrated in the left putamen, pallidum, caudate nucleus, and bilateral thalamus. The above regions let researchers distinguish IGD subjects from healthy controls [68].

A community structure (recruitment and integration) analysis of two networks, the executive control network (ECN) and the reward network, demonstrated that IGD males had a lower recruitment coefficient within the right ECN. The integration coefficient of the right ECN mediated the recruitment coefficient association of the right ECN and the reward network in recreational game users [69]. IGD individuals demonstrated lower functional connectivity in the ECN and raised connectivity in the reward network. NAcc–ECN functional connectivity was negatively correlated with NAcc–reward network functional connectivity [70]. Deficits in the ECN exhibited in the Stroop task [71,72] or discounting task [73,74] were found in other studies. The IGD group demonstrated an efficient and economic brain network, as well as a small-world topology (similar to the healthy controls). Yet the IGD individuals had decreased regional centralities in the PFC, left posterior cingulate cortex (PCC), right amygdala, and bilateral lingual gyrus [75]. The abovementioned observations indicate that IGD may be related to functional network dysfunction (executive control and emotional management). However, it is also associated with better coordination among visual, sensorimotor, auditory, and visuospatial systems (sensory motor regions functional connectivity) [75]. Another resting state connectivity study stresses the role of the dopaminergic system. Ventral tegmental area (VTA; one of the key nodes of mesolimbic dopamine pathways) circuits successfully identified IGD individuals. They mainly included the bilateral thalamus, right hippocampus, right pallidum, right temporal pole superior gyrus, and bilateral temporal superior gyrus [76]. IGD individuals had reduced functional connectivity between the VTA and right NAcc [77]. The functional and structural connectivity of the VTA–NAcc pathway and functional connectivity of the VTA–medial orbitofrontal cortex (mOFC) pathway were associated with IGD in other studies. It was also suggested that lower structural connectivity may underlie its vulnerability, while lower functional connectivity may underlie its severity [77,78].

Zhang et al. [79] support a triple-network model in IGD, which stresses that interactions between the executive control network/DMN and salience network might be important to understand the neural basis of IGD, as follows: the decreased modulation of the activity of the executive control network versus DMN by the salience network. They suggested that the resource allocation index [80] might serve as an indicator of altered brain activity in IGD patients (decreased in the right hemisphere). A narrative review by Mestre-Bach et al. [81] confirmed that most studies using independent component analysis found alterations in the abovementioned networks.

The insula’s role was examined by Zhang et al. [82]; IGD severity positively correlated with the anterior insula–angular gyrus–superior temporal gyrus functional connectivity and the posterior insula–superior temporal gyrus functional connectivity. The duration of gaming positively correlated with anterior insula–ACC functional connectivity. Also, higher insula activation was observed in persistent IGD individuals (compared to naturally recovered) during cue-craving tasks after forced gaming breaks [83]. According to Zhang et al. [84], an altered insula-based network is a neurobiological marker of IGD. IGD individuals demonstrated reduced functional connectivity between the left posterior insula and the bilateral SMA and MCC and between the right posterior insula and the right SFG. Decreased connectivity was observed between insular subregions.

IGD individuals, compared to non-IGD individuals, had a significant activation in the bilateral medial frontal gyrus, the left cingulate gyrus, the left medial temporal gyrus, and the fusiform gyrus. The time spent online correlated with activations in the left medial frontal gyrus and the right cingulate gyrus. The prefrontal dysfunction was proposed to be a good candidate for the IGD biomarker [85].

The shift from goal-directed to habitual systems in IGD was supported by a study on disturbed thalamocortical communication [86]. The study was based on the assumption that Internet-addicted individuals keep responding to learned reward stimuli, even when the expected outcome does not follow [87]. The brain region suggested to be responsible for the acquisition of goal-directed behavior and the depreciation of outcomes is the thalamus [88], and cortico-striato-thalamo-cortical circuits are the key to guiding behavior to acquire rewards and avoid punishments [89]. The study showed that IGD individuals had increased left midline nucleus (MN)–right PCG–pulvinar-medial frontal gyrus functional connectivity compared to RGUs [86]. Also, within the IGD group, a correlation between the correct response rates to inconsistent stimulus–result pairs and pulvinar–medial frontal gyrus connectivity was observed. Inhibition control scores correlated negatively with left MN–right PCG functional connectivity. Increased connectivity between the right thalamus and right PCG was observed both in IGD individuals and tobacco-addicted ones [90]. The initial phases of IGD were associated with increased cue-elicited (after a gaming session) activation of the bilateral lentiform nucleus. Recreational players who demonstrated such activation developed IGD a year later [91]. Wang et al. suggested that spectral dynamic causal modeling is more precise than resting-state functional connectivity in the neurobiological identification of IGD individuals [92]. They proposed the decreased excitatory mPFC-to-PCC connectivity as a crucial IGD biomarker. Another study suggests that cingulate–ventral striatal functional connectivity may serve as a self-control regulator in IGD [93].

A possible biomarker for the efficacy of psychobehavioral intervention was proposed by Zhang et al. [94]. The results showed that the therapy reduced the strength of resting-state VS–left inferior parietal lobule connectivity (mediating gaming craving and attentional bias). Before the intervention, the IGD group had higher VS-to-left inferior parietal lobule, rIFG, and left MFG connectivity. Wang et al. [95] pointed to the effective connectivity between the parahippocampal gyrus and the PFC as a possible biomarker of IGD. IGD subjects demonstrated lower effective connectivity from the left parahippocampal gyrus to the right MFG and from the right parahippocampal gyrus to the ACC, as well as reduced self-connection in the right parahippocampal gyrus. The most informative regions in the IGD-discriminating model (82.67% accuracy) were the bilateral parahippocampal gyrus, right ACC, and MFG [95]. A multi-voxel pattern analysis displayed an accuracy of 92.37%, sensitivity of 90.00%, and specificity of 94.74% in distinguishing IGD individuals from healthy ones. The regions that contributed most were the bilateral MFG, precuneus, and posterior lobe of the right cerebellum. The same study demonstrated that the craving behavioral intervention outcomes were best predicted by the right MFG, SFG, supramarginal gyrus, anterior/posterior lobes of the cerebellum, and left PCG [96]. Also, one year of cognitive behavior therapy increased cortical brain activity within the right MFG (all groups) and functional connectivity between the cortex and subcortex in groups with good prognosis (compared to groups with poor prognosis). Prior to the treatment, cortex–subcortex functional connectivity was decreased relative to healthy controls, right insular cortex activity correlated positively with playing time, and left MFG activity correlated negatively with IGD severity [97].

A meta-analysis of 25 studies on resting-state functional connectivity revealed that IGD individuals had low connectivity within the DMN and the following increased connectivity: DMN–insula within the ventral attention network, ventral attention network–somatomotor regions, and limbic network–regions of the frontoparietal network [98]. The authors’ conclusions are based on the interaction of the person–affect–cognition–execution (I-PACE) model [99] and the “learning to lose control” hypothesis [100], which stresses the transition between goal-directed and habitual behaviors. They suggest that the reinforcement of a reward system is a key functional biomarker for IGD. The frontoparietal areas have reward-related experience retrieval and reward anticipation functions and play an executive attention distribution role when an individual is confronted with reward stimuli. A recent study revealed that a possible biomarker of IGD addiction severity might be the decreased dynamics of the prefrontal–striatal and striatal–DMN neural circuits [101]. IGD subjects had reduced participation coefficients in the DMN and the frontal-parietal network during cue-craving tasks; a greater number of connections between nodes in the ventromedial PFC, ACC, and PCC was observed in addicted players compared to recreational ones [102]. Song et al. point to the DMN (resting state) as the most informative network in predicting IGD [103]. Also, it plays a crucial role in the craving network [104]. Increased functional connectivity within the DMN, as well as within the reward/salience network, was also observed in a resting-state EEG study [19].

#### 3.2.2. Ventral-to-Dorsal Striatal Shift

Some studies note that IGD may also be associated with dorsal striatum (DS) activity. Consistent with findings from substance use disorders [105,106,107], Liu et al. observed that the differences between IGD and healthy controls were more pronounced for the DS compared to the ventral one [108]. This would suggest the transition from ventral to dorsal striatal processing of addiction-related cues, which corresponds with the transition from social to heavy drinking [105]. Additionally, a negative association between subjective cue-induced craving and cue reactivity in the left VS was observed in IGD individuals. However, no corresponding association with DS was found. This might indicate a neurobiological difference between IGD and substance addictions. Zheng et al. point to the cerebellum as a region associated with craving dysfunction, both in substance and behavior addictions, yet the reward dysfunction in substance addictions is associated with the subcortical regions, and the reward dysfunction in behavioral addictions (IGD) is associated with the cortical regions [109].

The ventral-to-dorsal striatal shift was supported by evidence from a resting-state fMRI [110]. IGD individuals, compared to those using games recreationally, had lower VS connectivity with the middle frontal gyrus (MFG) (mainly in SMA) and higher DS (putamen) connectivity with the MFG. Also, this group showed a correlation between DS–MFG communication and DSM-5 scores. The study concluded that the severity of IGD is positively associated with left putamen–MFG connectivity and considered an imbalance within the frontostriatal circuits a potential addiction risk factor. However, slightly different results were demonstrated by Dong et al. [111]; the IGD group had lowered functional connectivity between the putamen and the SFG, MFG, and IFG, as well as between the VS and IFG, superior temporal gyrus, and MFG. Craving and addiction severity correlated negatively with the striatal–frontal regions’ functional connectivity.

Dorsal putamen functional connectivity was proposed as a potential IGD biomarker by Hong et al. [112]. Wang et al. [113] observed that in response to gaming cues, recreational gamers showed a positive correlation in the putamen–MFG–insula pathway, which was absent in the IGD group. Additionally, an inhibitory effective putamen–MFG connection in IGD individuals was negatively correlated with craving scores and was explained in terms of hypoactivation in the control system. The striatum–PFC pathway was suggested as a potential IGD risk biomarker. Wang et al. [114] suggest that the maintenance of IGD may be related to NAcc–insula and putamen–insula connectivity, which supports the ventral-to-dorsal striatal shift. An earlier study demonstrated that IGD was associated with lower left DLPFC–left insula, OFC–left insula, and higher insula–contralateral insula functional connectivity. The latter connectivity correlated positively with impulsivity. Additionally, lower functional connectivity was observed between the left DLPFC and left NAcc, between the left DLPFC and right NAcc, and between the insula and right NAcc. Higher functional connectivity was demonstrated between the right precuneus and the left or right NAcc [115]. A combined resting state and non-resting state study showed the functional connectivity of selected brain regions during and after imagery of gaming and alternative activities. The IGD group had raised caudate–right parahippocampal gyrus and putamen–right OFC connectivity during gaming that was reduced during alternative activities. The NAcc–right precuneus link was decreased at baseline, increased post-game, and unchanged post-alternative [116]. The static and dynamic functional connectivity of the DLPFC confirmed that it is one of the core regions underpinning IGD. IGD patients had their static functional connectivity reduced between the right DLPFC and the left Rolandic operculum and increased between the right DLPFC and the left pars triangularis. Dynamic functional connectivity was lower between the right DLPFC and left insula (a negative correlation with IGD severity) and the right putamen and left precentral gyrus, while it was raised in the left precuneus [117]. Whole-brain Granger causal connectivity (GCC) of the striatum and amygdala subdivisions showed that IGD individuals with high scores in behavioral inhibition and behavioral activation systems had reduced strength in the connectivity from the right MFG to the caudate, from the left SFG to the centromedial amygdala, and from the right superior parietal lobule to the left laterobasal amygdala, while in recreational players, the strength was raised from the left putamen to the right cuneus (IGD VS recreational gaming). Granger causal connectivity from the centromedial amygdala to the MFG mediated the directional relationship between behavioral inhibition and Internet addiction [118]. Disrupted functional connectivity of the amygdala may reflect impulsivity [119]. It was reduced between the left amygdala and left DLPFC, between the right amygdala and left DLPFC, and between the right amygdala and OFC. On the other hand, it was raised between the bilateral amygdala and the contralateral insula.

#### 3.2.3. GM Volume, Cortical Thickness, and Other Measurements

A longitudinal study showed that IGD may be associated with a smaller GM volume in the ACC and anterior-middle cingulate cortex (aMCC), decreased left dorsal putamen–left mPFC functional connectivity, and increased right dorsal putamen–right middle occipital gyrus connectivity, which supports the hypothesis of the weakening of prefrontal control and strengthening of the sensorimotor network [120]. Another longitudinal study [121] pointed to the role of the OFC. Initially, excessive players had lower right OFC GM volume (correlation with addiction severity) compared to gaming-naïve controls. After 6 weeks of gaming, the left OFC GM decreased in both groups (unlike in both groups under the non-gaming condition). In the IGD group, decreased GM volume was observed in the DLPFC, OFC, ACC, and right SMA. These regions showed lower functional connectivity with the insula, temporal, and occipital cortices, as well as other subcortical regions, as follows: DS, pallidum, and thalamus [122]. Lower right NAcc volume (compared to gaming controls but not healthy ones) and GM density in the DLPFC and IGD subjects were shown in another study [123]. A reduction in GM volume was demonstrated in the left ventrolateral PFC and the left IPL [124], as well as a reduction in GM density in the bilateral amygdala [119]. Higher left VS GM volume was observed in frequent video game players compared to infrequent ones [125]. High IGD-scoring individuals had increased GM volume in the right mPFC and precuneus [126]. The IGD group demonstrated negative cortical thickness deviation in the PCC, while TUD patients demonstrated a positive one [127].

IGD males had greater cortical thickness and IGD females had smaller cortical thickness in the bilateral rostral MFG, left SFG, left supramarginal gyrus, and right superior parietal lobule relative to same-sex recreational players. On the other hand, cortical thickness in the right PCC was reduced in males and increased in females. The authors suggested that males and females may be affected differently by IGD [128]. IGD individuals had reduced cortical thickness in the right rostral ACC, right lateral OFC, and left pars orbitalis and smaller GM volume in the right caudal ACC and left pars orbitalis. A whole-brain analysis showed lower thickness in the right SMA, left frontal eye field, superior parietal lobule, and PCC [129]. Another study demonstrated reduced cortical thickness in the left lateral OFC, IPL, bilateral cuneus, precentral gyrus, and right middle temporal gyrus, as well as decreased cortical volume in the left superior temporal gyrus and right supramarginal gyrus [130]. Finally, in IGD youths, cortical thickness was raised in the bilateral insulae and the right inferior temporal gyrus and reduced in the bilateral banks of the superior temporal sulci, the right inferior parietal cortex, the right precuneus, the right precentral gyrus, and the left middle temporal gyrus [131]. Higher GM volume was also observed in the bilateral caudate (dorsal anterior, body, tail) and the left NAcc, while a bigger white matter volume was reported in the inferior parietal lobule. However, there were no white matter volume alterations in the mesocorticolimbic dopaminergic system. GM volume in the left lateral orbital gyrus of the OFC correlated with game craving, and GM volume in the left anterior insula, right NAcc, right caudate, and right OFC correlated with self-control [132].

A study on the white matter network showed that IGD individuals had reduced global efficiency and local efficiency, as well as increased shortest path length. Nodal efficiency was decreased in the frontal cortex, ACC, and pallidum [133]. An earlier study associated gameplay with white matter connectivity. IGD subjects had increased myelination (higher fractional anisotropy and lower radial diffusivity values) in the right-sided frontal fiber tracts. IGD duration correlated positively with the integrity of white matter fibers and negatively with the diffusivity of axonal density (whole-brain white matter) [134]. Raised fractional anisotropy values were observed in in the bilateral anterior thalamic radiation, anterior limb of the internal capsule, bilateral corticospinal tract, bilateral inferior fronto-occipital fasciculus, corpus callosum, and bilateral inferior longitudinal fasciculus. Fractional anisotropy values correlated with IGD severity [135]. In general, GM volume in the prefrontal and striatal areas was proposed to be a structural biomarker of IGD and other behavioral addictions [136].

Another observation comes from a cognitive control resting-state study [137]. Increased right caudate and NAcc volume, as well as reduced connectivity strength of DLPFC–caudate and OFC–NAcc, was demonstrated in IGD subjects. The caudate volume and DLPFC–caudate connectivity correlated with more errors in the Stroop task. Also, individuals more preoccupied with games had higher left caudate nucleus–right DLPFC connectivity, and the right DLPFC and the right middle frontal cortex were positively correlated with IGD severity [138]. This corresponds with earlier findings that showed that IGD individuals had increased volume of DS (caudate) and VS (NAcc) and made more errors in the Stroop task. In this group, the Stroop task results correlated with caudate volume, and NAcc volume was related to Internet addiction scores [139]. IGD individuals (compared to RGUs) had inhibitory effective rOFC–right caudate (negative correlation with IGD severity) and right DLPFC–left OFC connectivity. Also, excitatory effective thalamus–left OFC connectivity was observed [140]. A radiomics-based machine learning model constructed with 20 features from the right caudate nucleus and amygdala successfully distinguished IGD patients from healthy controls [141]. Another diagnostic model included cortical alterations in the bilateral fusiform, left rostral middle frontal gyrus, left cuneus, left pars opercularis, and regions around the right uncinate fasciculus and left internal capsule [142]. There is some evidence that IGD may be better detected on the basis of system-level alterations than specific region features [143].

Brain controllability is proposed as another IGD biomarker. Two DMN components, the right PCC and the right orbital medial frontal cortex, were related to the level of addiction, and the left superior frontal cortex regulated the process. On the other hand, the biggest differences in modal and average controllability between the IGD group and the controls were observed in the inferior parietal cortex. In general, addicts had greater synchronizability and modal controllability compared to healthy individuals. This may help their brains to switch to the craving states and maintain them [144]. Another study shows that the right medial orbital part of the SFG could be attributed to the IGD biomarker base as an indicator of an impulsivity trait [145].

#### 3.2.4. IGD Subtypes

A recent study [146] gave evidence that IGD may be a heterogeneous disorder. The following two IGD subgroups were identified: craving-related (mostly males; high craving, fMRI cue reactivity, positive results of a craving behavioral therapy) and mixed psychological (mostly females; high craving, behavioral inhibition and activation scores, non-adaptive emotion regulation strategies and guessing-task fMRI measure). The first group demonstrated increased functional connectivity strength in brain networks (especially insula–PFC). The second showed decreased functional connectivity strength, especially involving the striatum–PFC and amygdala–PFC. This group’s IGD may involve weaker regulation of negative emotions. Zhou et al. [147] argued that gaming cues affect inhibition control more in males than in females, which makes it more difficult for them to give up addicted behavior. When exposed to gaming cues, males demonstrated lower activation in the ACC, SFG, and posterior cingulate cortex (PCC). Also, males showed higher middle temporal gyrus–PCC–right ACC/parahippocampal gyrus effective connectivity than females. Reduced activation of the ACC, PCC, and middle temporal gyrus was observed during risk-taking tasks in IGD males compared to healthy male controls [148]. Also, males showed higher middle temporal gyrus–PCC–right ACC/parahippocampal gyrus effective connectivity than females. Males seem to be more vulnerable to IGD [149]. Male IGD subjects demonstrated increased connections between the frontal-parietal network and cingulo-opercular network, reduced modular segregation of the frontoparietal network, and raised causal influence from the cingulo-opercular network to the frontal-parietal network (compared to male recreational players), which was not observed in female groups. The evidence for sex differences in IGD also comes from a non-resting state study [150]. Males, unlike females, had reduced DLPFC–SFG and increased striatum–thalamus functional connectivity during playing (IGD subjects compared to recreational players). After a forced break, DLPFC–SFG functional connectivity was decreased in both IGD males and females, and recreationally playing males showed weaker functional connectivity than corresponding females. Both IGD males and females had raised striatum–thalamus functional connectivity, yet the female groups demonstrated greater differences. Sex differences were also observed in an fMRI regional homogeneity study [151]; male IGD individuals showed raised regional homogeneity in the left MOG and the right middle temporal gyrus, while IGD females demonstrated lowered regional homogeneity in these regions (compared to the same-sex recreational players). Also, IGD males, unlike IGD females, had decreased regional homogeneity in the right PCC, which correlated negatively with Internet addiction scores. The opposite PCC result in the male population was given by a study comparing IGD, AUD, and healthy individuals [152]. Regional homogeneity was raised in the PCC in both patient groups. Also, decreased regional homogeneity was found in the right superior temporal gyrus in IGD subjects relative to other groups. The previous study’s controls were recreational players; this one, however, compared IGD persons to healthy ones. The ambiguous result may stem from different group selection and possibly indicates different correlates of IGD development phases.

The study on sex differences showed that IGD males, compared to healthy males, had decreased seed connectivity between the orbital part of the left SFG and PCC, the right angular gyrus, and the right DLPFC. Also, the amplitude of low-frequency fluctuation values in the orbital part of the left SFG was lower in the first group and correlated negatively with impulsiveness scale (behavioral inhibition trait) results. Such differences were not observed between IGD males and females and within the female groups. The authors conclude that in the male population, behavioral inhibition (crucial to IGD) could be biomarked by the altered amplitude of low-frequency fluctuation values in the orbital part of the left SFG [153].

#### 3.2.5. Synaptic Integrity, Dopamine, Glucose, and N-Acetylaspartate

A recent PET study proposed altered synaptic integrity as a possible IGD biomarker. Lower synaptic density in the right ACC, bilateral putamen, and right Rolandic operculum was found in IGD individuals, and synaptic density in the bilateral putamen correlated with IGD severity. Also, prolonged stop-signal reaction times were associated with synaptic vesicle glycoprotein 2A density in the right pregenual ACC and right Rolandic operculum. The observations may point to the role of inhibitory control in IGD [154].

Some IGD studies focus on dopamine release. Single-photon emission computed tomography showed no change in the levels of dopamine D2 receptor occupancy in the caudate in response to a motorbike riding game in ex-chronic “ecstasy” users compared to healthy individuals. The result was explained in terms of a sensitization mechanism [155]. The striatal function driven by dopamine is proposed as a core factor in promoting addictive behavior [125]. Non-addicted individuals had increased endogenous dopamine in the VS when playing a game [156]. IGD subjects had a significant decrease in glucose metabolism in the prefrontal, temporal, and limbic regions. The time of overuse was correlated with the dysregulation of D2 receptors in the striatum, and low levels of D2 receptors in the striatum were associated with decreased glucose metabolism in the OFC [157]. Another study [158] showed that IGD individuals had reduced fluoro-2-deoxyglucose uptake in the left medial orbitofrontal gyrus, left MCC, left SFG, and right ACC compared to healthy controls. Also, the regional cerebral metabolic rate of glucose correlated negatively with the number of fulfilled diagnostic IGD criteria. IGD individuals relative to healthy controls had raised levels of glutamate and glutamine (Glx) in the striatum [159]. Decreased levels of brain metabolite N-acetylaspartate in the right DLPFC were observed in the IGD group compared to the control group, which was predicted by ADHD history [160]. A similar result was demonstrated by Bae et al. in adolescents [161]. ADHD patients with or without comorbid IGD showed reduced N-acetylaspartate levels within the frontal cortex. Additionally, the Glx level was raised only in the non-IGD ADHD individuals, although it correlated positively with ADHD and inattention scores in the IGD+ADHD group.

A narrative review conducted by Carpita et al. [162] pointed to the role of dopamine in the striatum, which reduced the levels of dopamine D2 receptor availability [163] and reduced dopamine transporter availability [157]. The interaction of dopamine–glutamate is linked to drug-seeking behaviors [164] and reward processing in addiction [165].

### 3.3. Genetic Studies

There is evidence that Val/Val genotype carriers (catechol-O-methyltransferase (COMT) val158met polymorphism) may be more susceptible to IGD due to the fact that they catabolize several times more dopamine than carriers of the homozygous Met/Met variant [166]. Dopamine transporter level was associated with IGD depressive symptoms [167]. A search for genetic IGD markers revealed that 141C Ins/Del polymorphism in the male population may be a trait marker of potential IGD. In the IGD group, the del+ genotype was associated with higher novelty seeking [168]. IGD individuals also showed lower numbers of the T allele of rs1044396 in the nicotinic acetylcholine receptor alpha 4 subunit (CHRNA4), and this variant is associated with a protective effect against IGD [169]. Another protective role in IGD seems to have rs2229910 of neurotrophic tyrosine kinase receptor type 3 (0.1541 odds ratio) [170]. Also, the carriers of the TT genotype of rs1137070 were more prone to develop IGD than the C carriers (2.25 odds ratio) [171]. A much higher risk of IGD was observed in individuals with lower expressions of three miRNA alterations (hsa-miR-200c-3p, hsa-miR-26b-5p, hsa-miR-652-3p). The protein expression of GABRB2 and DPYSL2 was significantly higher in the IGD group. Circulating miRNAs were considered to be biomarkers for different psychiatric disorders (schizophrenia, major depressive disorder, Alzheimer’s disease) [172]. One study pointed out that the OXTR gene rs53576 polymorphism moderated a link between parent–adolescent conflict and IGD [173]. Another relevant study showed that the Taq1A1 allele of the dopamine D2 receptor and Val158Met in catecholamine-O-methyltransferase were more frequent in the excessive Internet video game play group than in the healthy group [174]. Also, similarly to depression, SS-5HTTLPR may be a harm-avoidance personality/environmental factor leading to IGD vulnerability in Internet addicts [175]. In IGD individuals, SS-5HTTLPR can be a risk factor for impulsiveness [176]. More specific information can be acquired from the Carpita et al. narrative study [162].

### 3.4. Blood Sampling

Serum levels of glutamate in young IGD males were lower compared to control subjects, while serum dopamine levels did not differ between groups, although serum dopamine levels were positively correlated with glutamate levels. The observations may suggest the co-transmission of glutamate and dopamine in IGD. The lack of correlation between serum glutamate levels with exposure duration and Internet gaming and gaming hours suggests that glutamate dysfunction may be a biomarker of the IGD early stage [177].

Mass spectrometry-based blood plasma metabolite profiling revealed that IGD was associated with metabolites (arabitol, myo-inositol, methionine, pyrrole-2-carboxylic acid, aspartic acid), which explained 66.3% of the IGD variation. The study showed that IGD has a different linear regression model than ADHD. The first one includes depression as a clinical parameter, and the latter includes anxiety [178]. IGD individuals had an elevated orexin A level compared to the healthy controls, and gaming time in the IGD group correlated negatively with the expression of brain-derived neurotrophic factor (BDNF) [179]. Raised serum levels of kynurenine and a reduced kynurenine acid/kynurenine ratio in both IGD and AUD individuals suggested stress-related psychoimmunological changes in these patients compared to the healthy controls [180]. Also, the IGD group exhibited higher levels of serotonin in serum samples [181]. Another possible candidate for an IGD biomarker comes from an integrative lipidomic profiles study. Two lysophosphatidylcholines (16:0 and 18:0) successfully discriminated IGD individuals from controls [182].

### 3.5. 2D:4D Digit Ratio

The index-to-ring finger length ratio is considered a noninvasive, indirect marker for prenatal testosterone exposure [183]. Males (females did not participate in the study) with high at-risk/addicted behavior had lower 2D:4D values than those with unproblematic video gaming behavior. The results were regarded as a proxy of the endophenotype “hyper-male brain organization” [184]. Different observation comes from the Müller et al. study [185]. A higher right-hand digit ratio (lower prenatal testosterone) was associated with higher IGD in the female group, but no such correlation was found in the male group. On the other hand, a meta-analysis study showed that a lower 2D:4D ratio was correlated with problematic substance and computer use, and this association was stronger for males than for females [186]. Considering a wide criticism of the use of 2D:4D as a biomarker of prenatal testosterone [186], further research in the IGD area is needed.

### 3.6. Arterial Blood Pressure/Heart Rate

A small group study demonstrated that instantaneous pulse rate variability (PRV) and instantaneous respiratory frequency can differentiate high-risk GD/Internet addiction individuals from low-risk ones while watching positive and negative game videos. Specifically, the high-risk group, compared to the low-risk group, showed higher normalized very high-frequency PRV and instantaneous respiratory frequency, as well as a lower normalized low-frequency and low-frequency/high-frequency PRV ratio [187]. Long et al. [188] demonstrated that high-risk players had higher low-frequency power/high-frequency power ratios compared to low-risk players throughout the early, middle, and late phases of game playing. They also had increased sympathetic activation in the early and late game phases [189].

Another study showed that IGD individuals had a reduction in high-frequency HRV during high-attention game playing and the last 5 min of the game relative to baseline values [190].

High-frequency HRV predicted IGD severity [191]. Similar results were observed in other studies [12,64,192]. Additionally, problematic Internet users (excessive gaming type) exhibited reduced cardiorespiratory coupling compared to healthy controls while playing an action video game. The result may indicate an altered central autonomic control over autonomic responses during pleasurable gaming [193]. The autonomic dysregulation hypothesis was also supported by resting state studies (Internet gaming addiction was associated with lowered HRV values [12,194]) and in the craving condition (a decrease in the standard deviation of the heart rate and an increase in the mean respiratory rate in mild to severe IGD participants [195]). After adjusting for depression, anxiety, and impulsivity, IGD (and AUD) patients had lower standard deviation of the normal-to-normal beat interval index compared to healthy subjects. This was correlated with the level of stress, resilience, and addiction severity [196]. Ono et al. [197] used a 24 h electrocardiogram to detect change and reactivity in HRV in excessive and non-excessive Internet game healthy controls. The study showed weak differences within high-frequency band power between the groups; the averaged high-frequency was higher in excessive players, which the authors attributed to possible depression and pre-Internet tendencies.

### 3.7. Other Measurements

As mentioned in the previous section, the multimodal biosignals study in craving/neutral conditions showed that also other measurements might serve as useful tools in detecting craving for games [195]. Paradoxically, the amplitude of the skin conductance did not significantly change in the craving condition, and it was attributed to the negative effects associated with withdrawal [198]. The possible markers of game craving might be the number of eye blinks and the distance of the saccadic movements (decreased during gameplay) [195]. Two studies demonstrated that high-IGD individuals compared to healthy controls had a lower correct rate of an eye-tracking anti-saccade task [199,200].

This category also includes biomarkers based on the integration of data from weak modalities. Multiple-kernel support vector machine IGD prediction had an accuracy of 86.5%, sensitivity of 89.3%, and specificity of 83.3% [201]. However, this prediction was not only based on biological features (EEG and PET) but also on ten combined clinical features (e.g., resilience or aggression psychometric scores). The study showed that these clinical variables had the highest contribution to the optimal prediction model. Another multimodal biomarker proposition (90% accuracy) included 13 physiological features from three modalities (vertical saccadic movement (electrooculogram), standard deviation of normal-to-normal intervals, and pNN50 (photoplethysmogram)) and prefrontal theta power, prefrontal alpha power, prefrontal delta/gamma ratio, prefrontal delta/beta ratio, prefrontal theta/beta ratio, prefrontal alpha/beta ratio, frontal alpha/beta ratio, parietal delta/gamma ratio, occipital delta/gamma ratio, and occipital theta/gamma ratio (EEG). The measurements—taken while participants watched neutral or gameplay videos—successfully distinguished IGD individuals from those who played games rarely or those who enjoyed and played games regularly but did not meet IGD diagnostic criteria. It is interesting that the proposed model did not show between-group differences in the self-reported craving scores [202].

### 3.8. Psychometric Markers

IGD can be diagnosed using psychometric markers. The instruments fall into the following two groups: (1) specifically measuring gaming addiction and (2) measuring Internet addiction. In the second case, additional criteria are used to distinguish IGD from different addictions. To ensure that findings relate to IGD-only addicts, cited studies also used nine DSM-5 diagnostic criteria [4] (applied by a psychiatrist or another professional), as well as strict weekly playing time/lifetime playing minimums. A psychometric score was essential in determining IGD severity.

The most often used psychometric tools were Young’s Internet Addiction Test (IAT or YIAT or Y-IAT) based on or translated from the original 20-item Young’s scale (113 uses) [203] and 26-item Chen (Chinese) Internet Addiction Scale (CIAS or CIAS-R) developed by Chen et al. (23 uses) [198,204]. Other tools include the Compulsive Internet Use Scale (CIUS; 14 items; 1 use) [205], IGD-Short Form Scale (IGDS-SF9 or IGDS9-SF; 9 items; 6 uses) [206,207], Skala zum Computerspielverhalten (CSV-S; 14 items; 2 uses) [208], Young’s Diagnostic Questionnaire for Internet Addiction test modified by Beard and Wolf (YDQ; 8 items; 4 uses) [209], problem video game playing scale (PVP; 9 items, 1 use) [210], DSM scale (9 items; 1 use) [211], IGD-20 Test (20 items, 1 use) [212], Korean Internet Addiction Proneness Scale (K-Scale or KS-A; 15 items; 3 uses) [213], Internet Gaming Disorder Questionnaire (IGDQ; 9 items, 3 uses) [4], Video Game Dependency Scale (CSAS II; 14 items; 1 use) [214], Short Version of Internet Addiction Test (s-IAT; 12 items; 1 use) [215], and Online Gaming Addiction Scale for Adolescents (20 items, 1 use). Additionally, most of the studies used nine DSM-5 diagnostic criteria [4]. Noteworthy is the fact that IAT psychometric diagnosis studies use different criteria for IGD versus recreational player/healthy control group assignment; in most cases, an IAT score of 50 (out of 100) classifies individuals as IGD, yet some studies make a division point at 60. Table 1 lists biological and psychometric markers found in the empirical studies cited in this article.

## 4. Discussion and Conclusions

IGD is a relatively new disorder. ICD included it officially in its 11th revision in 2018 under the name “gaming disorder” in the category of disorders due to addictive behaviors. The disorder included both online and offline variants (6C51). The DSM-5 introduced it in 2013 in Section III as requiring further study. This narrative review mainly focuses on Internet gaming, with only two articles related to the broader GD. Interestingly, the majority of the cited studies are based on Internet addiction psychometric tests, and the gaming type is identified using additional criteria (e.g., time spent on games vs. other online activities). Prior to formal acknowledgment of GD/IGD, it was investigated under the following different names: intensive/excessive/problematic game playing/Internet use. Biomarkers serve as useful objectively measurable tools to help clinicians diagnose either IGD or its risk. The studies presented in this paper focus on neurobiological mechanisms rather than precise clinical criteria. A few studies propose diagnostic (multimodal) models of IGD [92,95,96,103,141,143,201,202] or IGD-related gaming craving [104,195]. However, some of the predictions are based on comparison with recreational players [92,95,143,202] and some are based on comparison with healthy controls/potential recreational players [96,103,104,141,195,201], which makes the suggested biomarker usable in a less broad or broader context. For example, PCC regional homogeneity was either reduced [151] or increased [152] depending on the control group. Some harmless biological alterations may stem from gaming practice [sensory motor-related brain networks, 74]; some, however, reflect deficits in emotional and executive networks [38,99]. The large database of diverse neurological mechanisms and potential biomarkers makes their diagnostic application slightly confusing. For example, there is a standpoint that resting-state measures are more representative of long-term changes [63], and at the same time, game craving is better captured while using cue-related procedures.

Although there is a visible consensus related to general IGD neurological background (raised reward-seeking and decreased cognitive control), the multiplicity of research methods and procedures used makes their practical diagnostic application difficult. First, findings from studies with recreational controls should be replicated in those comparing IGD and healthy participants (and vice versa). Second, results should be confirmed in larger samples. Third, cultural differences should be examined. The majority of studies cited in this article were conducted in Asian populations; therefore, it is unknown if the findings are also applicable to other cultures. Fourth, more research should address female IGD to explore if it shares the same biological background. Finally, there is a concern related to IGD diagnosis. DSM-5 scores ≥5 did not let researchers differentiate IGD from recreational game use in multivariate pattern analysis (regional homogeneity or functional connectivity). It was possible when DSM-5 criteria were raised to ≥6 and both fMRI features were used [216]. A similar problem is associated with diagnostic IAT scores (≥50 or ≥60).

However, the biomarkers presented here can certainly be treated as a point of departure for future studies on IGD biomarkers, in which more precise than raised/decreased findings would be available.

The evidence presented in this article allows us to ask whether IGD should be treated as a homogeneous problem. Firstly, NAcc measurements may manifest addiction progression. IGD individuals who are older and have more gaming experience demonstrated NAcc volume similar to that of healthy controls, while in non-IGD players, it was raised [123]. Lower NAcc volume was observed in males in their 20s and 30s [123], while higher NAcc volume was observed in young students aged 16–22 [137] and 17.9 ± 0.9 [139]. Also, decreased NAcc volume may be compensated by its higher activation [51,54]. Secondly, different psychological backgrounds and sex may underlie different neural patterns. Increased insula–PFC functional connectivity seems to be characteristic of craving-related, mostly male addicts, while striatum–PFC and amygdala–PFC functional connectivity for compulsive and weakly regulated emotions were characteristic of mostly female addicts [146]. Several studies demonstrated differences between male and female IGD [128,141,150,151,153]. IGD’s neurological basis may also depend on gaming patterns (e.g., game genre) [21].

The two theories mentioned in this review, I-PACE [99] and the tripartite system model [38], point in general to gaming-related problems with salience and inhibition. The first dysfunction makes an individual more susceptible to game stimuli (raised reward seeking), and the second one negatively impacts resistance to these stimuli (decreased cognitive control).

IGD patients’ hypersensitivity to reward manifests in VS measurements [40,43,44,48,51,53,54,93,94,108,116,132,137,139,156]. However, there is also empirical support for a ventral-to-dorsal striatal shift [108,110,111,112,113,114,123]. VS may be responsible for learning the values of stimuli, and DS may be responsible for habitual action [110]. The role of VS vs. DS needs further research, in particular, in larger samples and with stricter psychometric IGD criteria. The deficits in executive control were demonstrated in PFC, especially DLPFC parameters [40,41,42,44,45,47,48,50,51,57,58,63,64,72,83,115,117,119,122,123,137,150,153,160]. Also, alterations in ACC reflect the cognitive control deficit. Its activity is higher or lower depending on the research procedure. IGD individuals had a higher ACC activation than healthy controls [51] or recreational players [57]. Other studies showed opposing results in comparison with healthy [148] or recreational controls [42,52]. This could be explained in terms of the differences in research tasks. In the first case, the participants only watched the stimuli or tried to regulate their cravings; in the latter, they performed a cue reactivity or risk-related task that might have differently affected their control resources. However, a decreased ACC GM volume finding was congruent in four cited studies [120,122,124,129]. Since ACC mediates cognitive influences on emotions [217], its role should be further explored in various method and procedure contexts.

Precuneus parameters may deliver another useful biomarker, although the findings cited in this review are contradictory. The precuneus is responsible for habitual behaviors in reaction to cue-related stimuli [59,218], as its higher GM volume [126], connectivity to the hippocampal gyrus, and activation positively correlated with IGD severity [61]. On the other hand, it demonstrated decreased activation during the same research procedure in IGD individuals compared to recreational players [52]. The inconsistency may be attributed to additional factors [61] or the control group (similar to NAcc inconsistency; 123). Also, the right precuneus exhibited increased functional connectivity with the NAcc [115]. Another contradictory finding relates to the putamen–MFG functional connectivity during the resting state. It is either decreased [111] or raised [110]. Both studies had a large number of participants (without depression) and recreational players as controls. The dorsal putamen’s functional connectivity was proposed as the IGD biomarker [112]. However, further studies related to this region are needed. Some studies point to the parahippocampal gyrus, which is responsible for memory encoding/decoding, learning from experience, and—in terms of addiction—game craving [42,219]. Its activation in IGD participants was lower than in recreational players in cue reactivity tasks after forced-break gaming, which may suggest putting less effort into the task [42] and possibly more habitual gaming. Wang et al. [95] proposed parahippocampal gyrus–PFC effective connectivity as a possible IGD biomarker. This is also mentioned in other studies [116,127,147]. In summary, it can be observed that the reduced PFC GM can be compensated by its high activity. However, the connection of PFC with reward under cortex nuclei seems to be dysregulated. In terms of the tripartite IGD model, insular activity demonstrates changes in interoceptive awareness, and frontal cortex–under cortex functional connectivity indicates dysregulation in reward and self-control systems.

Another group of biomarkers includes EEG parameters. Increased absolute delta [11] and reduced left frontal theta power [35] may be explained in the context of the diminished activity of higher-order arrangements characteristic to earlier life stages, deep sleep, or some pathological states [220,221]. Knyazev [221] points to the restorative functions of slow waves related to brain activity and autonomic functions synchronization (e.g., the increase in signal-to-noise ratios in synaptic consolidation, replenishment of the glycogen, and glucose homeostasis). In another publication [222], he argues that the delta amplitude is higher during craving (abstinence) and decreases upon actual reward. Increased theta [11] may mark a similar problem. Moreover, since it has not changed after SSRI treatment, it can be considered a possible IGD trait marker [11]. Slow waves in the wake state manifest impulsivity and intolerance of delayed rewards, primitive defensive mechanisms [221], and weaker behavioral control. On the other hand, IGD individuals showed higher intrahemispheric beta [22] and gamma coherence, even after successful SSRI treatment [17]. It may indicate hyperactivity of the sensory and excitatory systems. Also, the increased intrahemispheric coherence may compensate for hemispheric hypofunction. In the light of the tripartite neurocognitive model [38], impulsivity and inhibition functions may be marked by gamma oscillations (high alertness) and slow-wave oscillations (low alertness) [16]. Additionally, reduced P3 amplitudes [19,25,26,27,29] point to cognitive impairments and delayed N2 latency [34] to impulsivity traits. Decreased N1 was proposed to be a possible IGD trait biomarker since it did not correlate with addiction scores [25]. However, it should be remembered that the IGD neurophysiological framework may differ depending on game usage patterns and genre [21].

PET studies revealed a reduction in the binding of raclopride to D2 receptors and an increase in endogenous dopamine in the striatum while playing a game [156]. Disordered players had lower glucose metabolism in the PFC, OFC [157], left medial orbitofrontal gyrus, left MCC, left SFG, and right ACC [158]. The levels of Glx were reduced in the striatum [159]. They also exhibited lower synaptic density in the right ACC, bilateral putamen, and right Rolandic operculum [154]. The abovementioned observations account for dysfunctions in the dopaminergic system and may underlie disordered players’ problems with self-control and impulsivity.

Other biomarkers are suggested by genetic studies. However, they point to temperament traits that may potentially manifest in various disorders. IGD was associated with catechol-O-methyltransferase (COMT) val158met polymorphism, involved in frontal cortex dopamine functioning [166]. Val/Val genotype carriers catabolize three to four times more dopamine than the carriers of the Met/Met variant, which negatively impacts their dopaminergic stimulation of the postsynaptic neurons [166,223], increases impulsivity and fun-seeking, and eventually may lead to addiction or other dopamine-related problems. In a study on IGD and reward dependence, low activity (COMT^L^) alleles and Taq1A1 alleles of the dopamine D2 receptor were more frequent in excessive players compared to healthy controls [174]. Similarly, the −*141C DRD2 del*+ genotype, which alters striatal D2 receptor binding potential [224], may play a role in IGD thanks to a higher level of novelty seeking [168]. On the other hand, the nicotinic acetylcholine receptor alpha 4 subunit (CHRNA4; modulation of the dopaminergic pathway) seems to be a protective allele against IGD [169]. Another protective variant is rs2229910 of NTRK3 [170]. IGD could also be marked by altered microRNA expression. The expression of hsa-miR-200c-3p, hsa-miR-26b-5p, and hsa-miR-652-3p was lower, while the expression of two of their downstream genes (GABRB2 and DPYSL2) was higher in IGD individuals compared to controls [172].

A blood analysis showed decreased serum glutamate [177], myoinositol, arabitol [178], and kynurenine [180] levels, and increased orexin-A [179] and 5-hydroxytryptamine [181]. Two lysophosphatidylcholines (C16:0 and C18:0) were identified as discriminating IGD individuals from healthy ones [182]. Blood pressure and ECG research revealed that IGD risk was associated with an increased low-frequency/high-frequency power ratio (during playing a game) [188], increased normalized very high-frequency PRV and instantaneous respiratory frequency, as well as a decreased normalized low-frequency PRV and low-frequency/high-frequency PRV ratio (while watching negative or positive gameplay stimuli) [187]. High-frequency HRV [12,64,190,192] and cardiorespiratory coupling were reduced [193] while playing. These findings may suggest that game-playing is more habitual than goal-oriented [64]. Finally, a lower 2D:4D finger ratio in IGD individuals [184,185] points to the role of prenatal androgen exposure, and the number of eye blinks and saccadic eye movements while watching game stimuli may mark game craving [195,202]. A decreased correct rate of an eye-tracking anti-saccade task may indicate dysregulation in emotion and inhibition systems [199,200].

IGD shares biological similarities with other behavior and substance addictions and can be easily differentiated thanks to psychometric measures or self-report, yet the differences can also be marked by the following biological indicators: lower absolute delta [13], delta, and beta connectivity in the right hemisphere [22], lower N1 amplitudes [25], and higher intrahemispheric gamma coherence [16] (compared to AUD patients); higher dorsal frontostriatal circuit to thalamus effective connectivity and higher connections in the medial prefrontal cortex (compared to tobacco addicts) [109]; negative cortical thickness deviation in the PCC (positive in the TUD) [127]. Zheng et al. suggest that reward dysfunction in substance addictions is associated with the subcortical regions and reward dysfunction in behavioral addictions is associated with cortical regions [109]. Turel et al. [40] note that social media addicts have a dysregulated reward system and an intact prefrontal inhibition system, while IGD individuals suffer from both deficits.

It is important to distinguish between stable, trait-indicating biological parameters that predict personality/temperament addiction risk (e.g., impulsivity) from measures loosely associated with psychological conditions. For example, it is not clear if alterations in P3 amplitudes in IGD individuals during oddball tasks [24] should be considered a state or trait biomarker. The latter case may be investigated through longitudinal treatment studies (e.g., [11,17,114]). Also, this narrative review reports a standing expressed in some of the cited studies [110,113] that assumes that differences between disordered and recreational gamers may provide information about IGD trait biomarkers. However, it is not clear whether addict-specific markers stem from gaming engagement or are associated with pre-IGD conditions.

Biomarkers presented here differ in the context of their clinical applicability. Some of them require additional tools (response to gaming cues, Iowa gambling task, etc.) and some depend on a single measurement (e.g., resting-state EEG, fMRI, or blood sampling). Also, resting-state EEG allows for mobile and healthier testing than neuroimaging. The ease of diagnostic use may be an important factor in clinical practice.

Finally, IGD biomarkers can be informative in the light of corresponding addiction theories. The tripartite neurocognitive model [38] stresses the following three psychological areas: impulsiveness, reflexiveness, and interoceptive awareness. Any markers indicating changes in these systems may lead to the application of more patient-targeted therapies. The I-PACE theory [99] emphasizes a gradual shift from goal-directed to habitual behaviors, which may help in the prevention of individuals at risk (VS activity) and the diagnosis of addiction (DS activity). On the other hand, potential IGD sources (genes, temperament traits, comorbid problems and disorders) can be useful in early risk estimation.

### Limitations

There is an increasing number of studies on the biological correlates of IGD. We limited our Google Scholar search to articles published since 2021 due to the length of this paper. Google Scholar search was used to complement the current narrative review with the most recent findings not available in PubMed.

## Figures and Tables

**Table 1:** IGD biomarkers presented in empirical studies. Participant descriptions are as follows: IGD individuals/healthy participants/others if not stated otherwise. Numbers in brackets in psychometric markers list a number of items used. Findings relate to IGD group if not stated otherwise. Abbreviations: ↑—increase; ↓—decrease; 2D:4D—index to ring finger length ratio; ACC—anterior cingulate cortex; ACG—anterior cingulate gyrus; ADHD—attention-deficit/hyperactivity disorder; aMCC—anterior-middle cingulate cortex; AUD—alcohol use disorder; dACC—dorsal anterior cingulate cortex; BDNF—brain-derived neurotrophic factor; BGN—basal ganglia network; CIAS—Chen (Chinese) Internet addiction scale; CIUS—compulsive Internet use scale; CPL—characteristic path length; CSAS II—video game dependency scale; CSV-S—Skala zum Computerspielverhalten; CT—cortical thickness; DLPFC—dorsolateral prefrontal cortex; DMN—default mode network; DS—dorsal striatum; ECG—electrocardiography; ECN—executive control network; EEG—electroencephalography; ERP—event-related potential; fALFF—fractional amplitude of low-frequency fluctuation; fMRI—functional magnetic resonance imaging; GD—gaming disorder; GM—gray matter; HC—healthy control; HRV—heart rate variability; IAT—Internet addiction test; IFG—inferior frontal gyrus; IGD—Internet gaming disorder; IGDS-SF9 and IGDS9-SF—IGD-short-form scale; IGDQ—Internet gaming disorder questionnaire; IGT—Iowa gambling task; IPL—inferior parietal lobule; K-scale and KS-A—Korean Internet addiction proneness scale; LLP—late low potentials; MCC—middle cingulate cortex; MDD—major depressive disorder; MFG—middle frontal gyrus; MK-SVM—multiple-kernel support vector machine; MN—midline nucleus; MOG—middle occipital gyrus; mPFC—medial prefrontal cortex; NAcc—nucleus accumbens; OCD—obsessive–compulsive disorder; OFC—orbitofrontal cortex; PCC—posterior cingulate cortex; PCG—postcentral gyrus; PFC—prefrontal cortex; PRV—pulse rate variability; PVP—problem video game playing; RGU—recreational game user; ReHo—regional homogeneity; RSN—reward/salience network; s-IAT—short version of Internet addiction test; SFG—superior frontal gyrus; SMA—supplementary motor area; SSRI—selective serotonin reuptake inhibitors; SUD—substance use disorder; TUD—tobacco use disorder; WLEIS—Wong and Law emotional intelligence scale; WM—white matter; vmPFC—ventromedial prefrontal cortex; VS—ventral striatum; VTA—ventral tegmental area; YDQ—Young’s diagnostic questionnaire for Internet addiction.

Authors	Participants	Psychometric Markers	Method	Findings	Conclusions
Kim et al. [11]	20/29	IAT (20)	resting-state EEG (measurement pre- and post-SSRI treatment)	↑absolute delta (total brain) and theta (central region) (baseline) delta band normalized after pharmacotherapy and correlated with improvements in IGD symptoms ↑absolute theta (baseline) predicted improvement	slow-wave activity may be an IGD marker increased theta at baseline may be a prognostic IGD marker
Park et al. [12]	53/58	IAT (20)	resting-state EEG HRV	↑theta band CPL negative correlation between standard deviation of normal-to-normal interval index and theta and delta CPL ↑HR ↓high-frequency HRV	IGD related to maladaptive brain–body integration features IGD related to suppression of parasympathetic and/or vagal tone
Son et al. [13]	34/25/17AUD	IAT (20)	resting-state EEG	↓absolute beta ↓absolute delta (compared to AUD)	absolute beta power may be IGD trait marker
Pandey and Jain [14]	databases (IGD and AUD)	-	resting-state EEG	↓absolute theta and beta in frontal and prefrontal regions (similar to AUD)	emotional imbalance, high arousal, slow response inhibition suggest similarity to AUD
Park et al. [15]	16ADHD+IGD/15ADHD/15	IAT (20)	resting-state EEG	↓relative delta in temporal regions in ADHD+IGD (compared to ADHD) ↑relative beta in temporal regions in ADHD+IGD (compared to ADHD) ↑intrahemispheric coherence values for delta, theta, alpha, and beta between P4 and O2 in ADHD+IGD (compared to ADHD) ↑intrahemispheric coherence values for theta between Fz–Cz and T4–T6 in ADHD+IGD (compared to ADHD)	greater vulnerability to attention difficulties seems to be correlated with Internet gameplay to enhance attentional ability
Park et al. [16]	30/32/30AUD	IAT (20)	resting-state EEG	↑intrahemispheric gamma coherence (while controlling for psychological characteristics) right frontocentral gamma coherence positively predicted Internet addiction scores (all groups)	phasic synchrony of gamma coherence may indicate IGD
Park et al. [17]	30/32	IAT (20)	resting-state EEG (measurement pre- and post-SSRI treatment)	↑intrahemispheric beta and gamma coherence (baseline) ↑right delta hemisphere intrahemispheric coherence (baseline) ↑intrahemispheric beta and gamma coherence after treatment despite symptom improvement no significant coherence changes after treatment	intrahemispheric fast-frequency coherence may be an IGD trait marker
Youh et al. [18]	14 MDD+IGD/15 MDD	IAT (20)	resting-state EEG	↓interhemispheric alpha coherence between Fp1 and Fp2 (MDD+IGD compared to MDD only) ↑intrahemispheric alpha coherence between P3 and O1 (MDD+IGD compared to MDD only) ↑intrahemispheric beta coherence among F8–T4, T6–O2, and P4–O2 (MDD+IGD compared to MDD only)	intrahemispheric coherence may stem from high gaming engagement
Lee et al. [19]	79/79	IAT (20)	resting-state EEG	↑theta, alpha, and beta functional connectivity between OFC and parietal regions ↑alpha and beta functional connectivity between the ACG and temporal regions negative correlation between severity of IGD symptoms and/or weekly gaming time and theta and alpha connectivity within DMN and theta, alpha, and beta connectivity within the RSN	increased connectivities within DMN and RSN may be potential IGD state markers
Lee et al. [20]	36/35	IAT (20)	resting-state EEG	↑alpha coherence in the right hemisphere (low-resilience IGD compared to high-resilience IGD and Hcs) resilience moderated indirect effects of IGD on alpha coherence through depressive symptoms and stress level	alpha coherence in the right hemisphere is a distinct feature associated with resilience in IGD high alpha coherence in the right hemisphere may be an IGD biomarker in low-resilience IGD subgroup only
Hong et al. [21]	76/64	IAT (20)	resting-state EEG	↑beta activity (single-game IGD players compared to multiple-game IGD players and HCs) ↑delta power in frontal region (first-person shooter players compared to Hcs) ↓theta intrahemispheric coherence in left frontoparietal region (multiplayer online role-playing game and first-person shooter players compared to HCs) ↓delta intrahemispheric coherence in left frontoparietal region (multiplayer online role-playing game players compared to HCs) ↓beta intrahemispheric coherence in left frontoparietal region (first-person shooter players compared to Hcs)	IGD neurophysiological networks may differ depending on game usage patterns and genre
Lee et al. [22]	67/66/58 AUD	IAT (20) (modified for games)	resting-state EEG	↓delta connectivity between right OFC and right angular gyrus and inferior parietal lobe in DMN ↑beta connectivity between right PFC and right temporal lobe in RSN ↑delta connectivity between right temporal lobe and the right PCC and cuneus ↓beta connectivity between the right PFC and the right ACC	IGD similar to AUD changes in delta and beta source-level functional connectivity (right hemisphere) distinguish IGD from AUD
Park et al. [24]	26/23	IAT (20) (modified for games)	EEG (ERP, oddball task)	↓response to deviant tones (P3 amplitudes at the midline centroparietal regions) negative IGD severity–P3 amplitude correlation	reduced P3 amplitudes reflect dysfunction in auditory information processing and cognitive capabilities in IGD
Park et al. [25]	26/29/22 AUD	IAT (20)	EEG (ERP, oddball task)	↓P3 amplitudes (midline central and parietal area; IGD and AUD) ↓N1 amplitudes at the midline frontal area (IGD) P3 correlated with higher spatial span error rate (IGD) N1 and P3 did not correlate with Internet addiction severity scores (IGD)	P3 similar in IGD and AUD N1 may be an IGD trait biomarker
Park et al. [26]	26/27	IAT (20)	resting-state fMRI EEG (ERP, oddball task)	↓ReHo and fALFF values in left inferior occipital gyrus ↑ReHo and amplitude of low-frequency fluctuation values in right precuneus ↑amplitude of low-frequency fluctuation in left SFG ↓P3 amplitudes in midline centroparietal area regional activity in right inferior temporal gyrus and occipital regions positively correlated with P3 amplitudes ReHo values of left hippocampus and right amygdala negatively correlated with P3 amplitudes	IGD related to impairment in interacting efficiency with cognitive function and sensory processing
Duven et al. [27]	15/15 casual computer gaming	CSV-S (14)	EEG (while playing a game)	↓P3 amplitude in response to rewards ↑N1 latency ↑increased N1 amplitude	game tolerance observed in IGD initial orienting toward reward consumes more capacity
Lin et al. [28]	26/26	IAT (20)	resting-state fMRI	↓fALFF values in cerebellum posterior lobe ↑fALFF values in superior temporal gyrus frequency band/group interactions in cerebellum, ACC, lingual gyrus, middle temporal gyrus, MFG	IGD related to abnormal fALFF values in many regions
Zhou et al. [29]	24/26 RGU	IAT (20)	EEG (ERP, decision-making)	↓P2 amplitudes during discount processing ↓P3 amplitudes in short- and long-term delays ↑N2 amplitudes for risk disadvantageous in the loss domain than the gain domain in RGU; no difference in IGD individuals	no difference between IGD and RGU subjects in impulsive and control systems IGD related to altered realistic reward preference, reduced loss aversion, and less effortful decision assessment
Kim et al. [30]	20/23/20OCD	IAT (20)	EEG (ERP, craving cues)	↑LLP amplitudes for game-related cues (compared to HCs)	LLP may be a biomarker for cue-related craving
Ha et al. [31]	20/20	IAT (20)	EEG (ERP, craving cues)	↑parieto-occipital theta power (IGD group; favorite compared to non-favorite game) parieto-occipital theta power correlated positively with self-reported craving (favorite game only)	higher parieto-occipital theta power in response to favorite game cues may mark IGD
Park et al. [32]	21/21	IAT (20)	resting-state EEG	↑absolute powers of central and parieto-occipital delta, theta, and beta	absolute powers of central and parieto-occipital delta, theta, and beta may help distinguish between IGD individuals and HCs
Kim et al. [34]	27/26/24OCD	IAT (20)	EEG (ERP, go/no-go task)	delayed no-go-N2 latency at the central electrode site (compared to HCs) positive correlation between IGD severity, impulsivity, and delayed no-go-N2 latency at the central electrode site ↑no-go-N2 amplitude at the frontal electrode site (compared to OCD)	prolonged no-go-N2 latency may serve as an impulsivity trait marker in IGD reduced no-go-N2 amplitude may differentiate OCD from IGD in context of compulsivity
Kim et al. [35]	24/35	IAT (20)	EEG (playing favorite game)	↓left frontal theta, alpha, and beta activities negative correlation between left frontal theta power and IGD severity	left frontal theta power may be a biomarker of diminished cognitive control patterns in IGD
Kim et al. [36]	15/15	IAT (20) CIUS (14)	EEG (playing League of Legends)	↓relative alpha and sensory motor rhythm ↑relative midbeta and highbeta beta relative can be ↑ or ↓ depending on which frequency of the beta is analyzed crossing phenomenon located between sensory motor rhythm (12–15 Hz) and mid-beta band (15–20 Hz)	right and left lobes respond differently (desynchronization may be an IGD biomarker) parameters from alpha, sensory motor rhythm, and mid-beta (Fp1, C3, C4, and O1) may be biomarkers of IGD symptoms
Park et al. [37]	33/31	IAT (20)	EEG (resting-state and ERP, go/no-go task)	negative correlation between alpha coherence at left frontocentral and bilateral centrotemporal electrodes with P3 latency (IGD) positive correlation between alpha coherence at left frontocentral and bilateral centrotemporal electrodes with P3 latency (HCs)	IGD lacks dynamic interactions of functional connectivity between the fronto-centro-temporal regions during resting state and ERP during cognitive tasks resting-state EEG and task-related ERP underlie IGD
Turel et al. [40]	26 intensive players/26	IGDS-SF9 (9)	fMRI (between-subjects factor (gamers vs. controls)), and 2 within-subjects factors: stimuli (game vs. neutral; all participants) and session (deprivation vs. satiety; only gamers)	IGD score positive correlation with activity in right VS IGD score negative correlation with DLPFC ↑VS activation in response to game cues ↓left frontal pole and DLPFC activation in response to game cues ↑left insular cortex activity under deprivation and with game cues (compared to other conditions) ↑right striatum activity during deprivation (compared to satiety) and with game cues than in satiety ↓bilateral DLPFC activity during satiety (compared to deprivation) and with game cues ↑left insula–left VS coupling with game cues (compared to non-game ones) during deprivation (but not during satiety) ↓left insula–left DLPFC coupling with game cues during deprivation (compared to non-game ones) during deprivation (but not during satiety)	game cues drive weaker prefrontal activation in gamers (gaming sensitizes the brain; it becomes automatic and dissociated from long-term motives) dysregulation deficits in reward and self-control systems (in social media addiction prefrontal-inhibition system intact) insula plays similar role in IGD and other addictive behaviors (cues under deprivation)
Dong et al. [41]	49 (27 male; 22 female)/50 RGU (27 male, 23 female)	IAT (20)	fMRI (cue-elicited stimuli; pre-game/post-game deprivation)	↓DLPFC activation in pre–post gaming tests (especially women) ↑caudate activation (post-game; IGD females compared to non-IGD females)	women with RGU may have better executive control than men when facing gaming cues
Zhang et al. [42]	49/49 RGU	IAT (20)	fMRI (after gaming forced break)	↓ACC, parahippocampal gyrus, DLPFC activation gaming craving correlated negatively with baseline activation level (bate value) of ACC, DLPFC, and parahippocampal gyrus	IGD related to control deficits (supressing gaming craving)
Liu et al. [43]	28/26	IAT (20)	fMRI (risk-taking task)	↑regret for significant missed chance ↑risk-taking ↑VS and SFG activation for significant missed chance ↑VS-thalamus connectivity for significant missed chance SFG activation—risk-taking correlation	neural responses to missed chance help explain IGD (continuation of playing despite negative consequences)
Liu et al. [44]	41/27	CIAS (26)	fMRI (cups task)	↓modulation for experienced risk within DLPFC and IPL for potential losses negative correlation between IGD scores and modulation of left DLPFC/bilateral IPL (IGD only) ↑responses for the experienced reward (potential gain) within VS, vmPFC, and OFC positive correlation between right OFC activity and IGD scores (IGD only)	imbalance between hypersensitivity for reward and weaker risk experience and self-control for loss
Lei et al. [45]	45/42	IAT (20) IGDS9-SF (9)	fMRI (reward-related prediction error task)	blunted reward prediction error in right caudate, left OFC, and right DLPFC ↑right caudate, right putamen, bilateral DLPFC, and right dACC connectivity right DLPFC and right dACC connectivity predicts variation of reward prediction error signals in left OFC	IGD associated with disrupted reward prediction error signaling and increased connectivity between brain reward system regions
Hong et al. [46]	27/22	-	fMRI (IGT)	↓IGT score no difference between groups in node-centric functional connectivity loss aversion correlated negatively with promoted bottom-up neuromodulation from right hippocampus to left IFG loss aversion in HCs correlated positively with edge community profile similarity of the edge between left IFG and right hippocampus at right caudate (suppressed by response consistency in IGD group)	reduced loss aversion in value-based decision-making and their related edge-centric functional connectivity help (similarly to other addictions) understand IGD mechanism
Wang et al. [47]	18/21	IAT (20)	fMRI (delay discounting task)	↓DLPFC and bilateral IFG activation ↑discount rate k discount rate k correlated positively with IGD severity	IGD associated with pursuit of immediate satisfaction
Shin et al. [48]	20/21	IAT (20)	FMRI (emotional go/no-go task)	↑DLPFC and VS activation (high-demand task) ↑dorsomedial PFC activation (high-demand task)	IGD associated with failure of response inhibition and dysfunction in inhibitory control network
Ko et al. [49]	26/23	CIAS (26)	fMRI (go/no-go task)	↑impulsivity ↑left orbital frontal lobe and bilateral caudate nucleus activation (when inhibiting response) ↓right insula activation	impaired insular function in error processing and higher frontostriatal network activation (to maintain inhibition performance) response inhibition (frontostriatal network) and salience network (ACC and insula) contribute to error processing
Chen et al. [50]	15/15	CIAS (26)	fMRI (go/no-go task)	↓right SMA/pre-SMA activity ↑impulsivity ↑SMA, DLPFC, caudate activation (HCs compared to IGD)	dysfunctional activation of the SMA for response inhibition may be a biomarker of IGD
Ko et al. [51]	10/10	CIAS (26)	fMRI (cue-related and neutral stimuli)	↑right OFC, right NAcc, bilateral ACC, medial frontal cortex, right DLPFC, right caudate nucleus activation the above activation correlated with self-reported gaming urge and game recalling	neural substrate of cue-induced craving similar in IGD and substance addictions
Wang et al. [52]	30/40 RGU	IAT (20)	fMRI (cue-related and neutral stimuli)	↑left OFC ↓activation of right ACC, right precuneus, left precentral gyrus, right PCG	IGD related to reward evaluation (OFC), executive control (inhibition; ACC), and ignoring game stimuli (precuneus, precentral, and postcentral gyrus)
Zuo et al. [53]	41/44	IGD-20 Test (20)	fMRI (two-armed bandit task)	↑activation of pre-SMA and VS (decision-making and estimation phases) ↓activation of PFC (during exploitative strategies) ↑exploitative strategies in decision-making	IGD related to increased reward-seeking and reduced cognitive control
Wang et al. [54]	27/26	IAT (20)	fMRI (roulette task)	↑risky behaviors ↑NAcc and caudate activation during reward anticipation and outcome monitoring (not during the choice evaluation)	IGD associated with oversensitivity of reward system to potential and positive rewards IGD individuals take more risk than HCs, although they are able to properly assess the risk values and correctness of decisions
Lee et al. [55]	24/24	K-Scale (15) IAT (20)	fMRI (risky decision-making)	↓dorsal attention network and anterior insular cortex activation OFC activation correlated positively with game-craving	IGD related to vulnerability when undertaking new behavioral strategies in high-risk situations
Ma et al. [56]	29/23	CIAS (26)	fMRI (cue-related and neutral stimuli)	positive correlation with IGD severity: temporo-occipital and frontoparietal networks negative correlation with game craving: game-control disengagement of temporo-insula network 4 functional networks different in IGD and HCs: temporo-occipital, temporo-insula, frontoparietal, dorsal-limbic	IGD related to substance addictions (cue reactivity)
Zhang et al. [57]	21/23 RGU	IAT (20)	fMRI (regulation of craving task)	↑right ACC, PCC, PFC, middle temporal gyrus, left DLPFC, and thalamus activation ↓right PCC–right IPL functional connectivity	deficits of craving regulation in IGD associated with the imbalanced coordination between the reward network and the executive network.
Du et al. [58]	27/35 (male adolescents)	IAT (20)	resting-state fMRI	↑global/long-range functional connectivity density in bilateral DLPFC and right inferior temporal cortex/fusiform ↑global/long-range functional connectivity density of bilateral DLPFC correlated positively with Internet addiction and behavioral performance	increased functional connectivity density may reflect a compensatory mechanism for maintaining behavioral performance
Zhou et al. [59]	21/23 RGU	IAT (20)	fMRI (game-related and food-related stimuli)	↑precuneus–caudate functional connectivity for game-related than food-related cues ↓precuneus–caudate functional connectivity for game-related than food-related cues (RGUs) precuneus–caudate functional connectivity positively correlated with game-craving scores precuneus–caudate functional connectivity positively correlated with food-craving scores (RGUs)	imbalance in primary/secondary reward sensitivities in IGD underlie game craving
Yu et al. [60]	37 excessive games/67 gaming-naïve controls	WOW gaming addiction scale	fMRI (cue-related and neutral stimuli; baseline and after 6-week gaming)	↑valence attribution and neural reactivity in PCC/precuneus (baseline) valence ↑valence rating and neural reactivity in PCC/precuneus (follow-up; gaming-naïve controls)	increased neural reactivity in PCC observable during early stages of regular gaming
Dong et al. [61]	65	IAT (20)	fMRI (cue reactivity task)	positive correlations with IGD severity: volume of precuneus precuneus activation hippocampal gyrus–precuneus functional connectivity negative correlation with IGD severity: MFG–precuneus functional connectivity	precuneus may help integrate potential contradictory information between executive control and subcortical cravings
Dong et al. [62]	27/43 RGU	IAT (20)	fMRI (cue reactivity task pre- and post-gaming)	↑PFC, striatum, precuneus activation (exposition to gaming-related stimuli; post- vs. pre-gaming) no differences in RGUs	gaming-enhanced activations in craving-related area
Liu et al. [63]	60 (after one year, 19 IGD recovered, 23 IGD persistent, 18 left the study)	IAT (20)	resting-state fMRI (60 participants)	↓OFC, precuneus, and DLPFC activity (persistent IGD compared to recovered IGD) increased ReHo values in OFC and precuneus (persistent IGD compared to recovered IGD)	reward processing and inhibitory control impairment (OFC, precuneus, DLPFC) are associated with development and maintenance of addictions
Lee et al. [64]	33/29	IAT (20)	resting-state fMRI HRV (during playing a game)	↓right DLPFC–right IFG functional connectivity ↓right ACC–superior parietal lobule functional connectivity ↑left dorsal putamen–PCG functional connectivity ↓high-frequency HRV during game-playing high-frequency HRV correlated positively with DLPFC–IFG functional connectivity no differences in GM volume	IGD related to worse cognitive control IGD game-playing is more habitual than goal-oriented
Lee et al. [65]	44 (childhood ADHD: 20; no childhood ADHD: 24/19)	IAT (20)	resting-state fMRI	↑DMN-related regions (PCC, mPFC, thalamus) functional connectivity (IGD/no ADHD compared to Hcs) ↑PCC–anterior insula, OFC functional connectivity (IGD/no ADHD compared to IGD/ADHD) ↑PCC–cerebellum functional connectivity (IGD/ADHD) PCC–cerebellum functional correlated positively with impulsiveness	altered neural networks for executive control in ADHD would be a predisposition for developing IGD (PCC functional connectivity)
Kim et al. [66]	94 (46 with ADHD, 48 without ADHD)/34	IAT (20)	MRI	IGD with comorbid ADHD have different network-level connectivity than IGD without ADHD edges connecting left precentral gyrus, left PCG, bilateral SFG, medial orbital parts, and left fusiform to the inferior temporal gyrus were best IGD predictors	aberrant increase in some structural connections (inhibitory function, sensory integration) indicates how comorbid ADHD is associated with IGD severity
Ye et al. [67]	402 (different IGD severity)	IAT (20)	resting-state fMRI	right precentral gyrus and left PCG most informative in prediction of IGD severity effective connectivity from right precentral gyrus to left precentral gyrus and dorsal ACC (positive correlation with IGD severity)	ReHo and ALFF in resting state can independently predict IGD severity
Niu et al. [68]	36/44	IAT (20)	resting-state fMRI	↑static and dynamic ReHo in the bilateral medial SFG, SFG, SMA ↑dynamic ReHo values in left putamen, pallidum, caudate nucleus, bilateral thalamus altered static and dynamic ReHo values let researchers distinguish IGD individuals from Hcs static ReHo values in left medial SFG, SMA, and dynamic ReHo values in left SMA correlated positively with Internet addiction dynamic ReHo values in left caudate nucleus correlated negatively with sleep quality	IGD related to impaired intrinsic local connectivity in frontostriatothalamic circuitry
Wang et al. [69]	162/240 RGU	IAT (20)	resting-state fMRI	↓recruitment coefficient within right ECN (IGD males) integration coefficient of right ECN mediated the relationship between the recruitment coefficients of right ECN and BGN (RGU group)	impaired impulse control in IGD top-down executive control of the ECN is absent in subjects with IGDk
Dong et al. [70]	35/36	IAT (20)	resting-state fMRI	↓functional connectivity in ECN ↑functional connectivity in reward network NAcc–ECN functional connectivity negatively correlated with NAcc–reward network functional connectivity	impairments in executive control lead to inefficient inhibition of enhanced cravings
Wang et al. [71]	18/19	IAT (20)	fMRI (Stroop task)	↑functional connectivity in middle temporal gyrus, superior temporal gyrus, MFG ↓functional connectivity in PCC, middle temporal gyrus, MFG	IGD related to abnormal right ECN functional connectivity
Dong et al. [72]	35/36	IAT (20)	resting-state fMRI Stroop task	↓functional connectivity in ECNs ↑activations in bilateral SFG ↓activations in DLPFC, ventral ACC, left OFC functional connectivity in ECNs correlated negatively with Stroop effect functional connectivity in ECNs correlated positively with activations in executive control regions	IGD related to impaired executive control (lower functional connectivity in ECNs)
Wang et al. [73]	19/21	IAT (20)	fMRI (probability discounting task)	↑activity in DMN ↓activity in ECN DMN activity correlated negatively with reaction ECN activity correlated positively with probability discounting rates	IGD related to deficit in executive control function
Wang et al. [74]	18/21	IAT (20)	fMRI (delay discounting task)	↑delay discounting rates ECN (ACC, MFG, SFG) and BGN (lentiform nucleus) associated with IGD ↑functional connectivity (selecting small and new options) delay discounting rates positively correlated with modulation of ECN and GGN and reaction time	IGD related to increased sensitivity to reward and decreased ability to control impulsivity
Wang et al. [75]	37/35	IAT (20)	resting-state fMRI	no differences in global topology metrics ↓regional centralities in PFC, left PCC, right amygdala, bilateral lingual gyrus ↑sensory motor-related brain networks functional connectivity (13 nodes in parietal, occipital, temporal lobes)	IGD related to dysfunctions in executive control and emotional management IGD related to better coordination among visual, sensorimotor, auditory, and visuospatial systems
Wen et al. [76]	66/80 + 14/14	IAT (20)	resting-state fMRI	VTA functional connectivity circuits identified IGD individuals (distinguishing features: bilateral thalamus, right hippocampus, right pallidum, right temporal pole superior gyrus, and bilateral temporal superior gyrus)	Resting-state VTA functional connectivity may bring IGD biomarker in the future
Zhang et al. [77]	35/24	CIAS (26)	resting-state fMRI	↓VTA–right NAcc functional connectivity VTA–right NAcc functional connectivity correlated negatively with Internet craving	VTA–right NAcc functional connectivity similar in IGD and substance addictions
Wang et al. [78]	33/28	IAT (20)	resting-state fMRI	↓NAcc and medial OFC–VTA functional connectivity strength of VTA–right NAcc and VTA–left medial OFC functional connectivity correlated negatively with Internet addiction (IGD group) ↓structural connectivity in bilateral VTA–NAcc tracts (no correlation with Internet addiction)	lower structural connectivity might underlie vulnerability to IGD lower functional connectivity may modulate severity of IGD dopamine reward system involved in IGD (VTA–NAcc structural connectivity and VTA–medial OFC functional connectivity)
Zhang et al. [79]	39/34	CIAS (26)	resting-state fMRI	↑salience network–DMN functional connectivity ↓resource allocation index in right hemisphere resource allocation index in right hemisphere negatively correlated with craving scores (IGD)	support for triple-network IGD model deficient modulation of ECN versus DMN by salience network frames neural basis of IGD
Zhang et al. [82]	74/41	CIAS (26)	resting-state fMRI	↑anterior insula–a network of regions, including ACC, putamen, angular gyrus, and precuneus, functional connectivity ↑posterior insula–PCG, precentral gyrus, SMA, and superior temporal gyrus functional connectivity IGD severity positively correlated with anterior insula–angular gyrus and superior temporal gyrus functional connectivity and posterior insula–superior temporal gyrus functional connectivity duration of gaming positively correlated with anterior insula–ACC functional connectivity	insula manifests core IGD symptoms
Dong et al. [83]	79/92 RGU (first measurement); 20 IGD persistent/20 IGD naturally recovered/40 RGU (measured 1 year later)	IAT (20)	fMRI (cue-craving task pre- and post-gaming)	↓DLPFC activation (IGD recovered compared to IGD persistent; pre- and post-gaming) ↓DLPFC and ↑insula activation (IGD persistent post-gaming compared to pre-gaming)	insula is a key neural structure in interoceptive processes
Zhang et al. [84]	19/19	IAT (20)	resting-state fMRI	↓functional connectivity between left posterior insula and bilateral SMA ↓functional connectivity between left posterior insula and MCC ↓functional connectivity between right posterior insula and right SFG ↓functional connectivity between insular subregions (posterior, ventroanterior, dorsoanterior)	IGD associated with altered insula-based network functional connectivity between interoception and motor/executive control regions reflects reduced ability to inhibit motor responses or diminished executive control over gaming craving
Zhou et al. [86]	25/25 RGU	IAT (20)	resting-state fMRI	↑left MN–right PCG functional connectivity ↑pulvinar–medial frontal gyrus functional connectivity pulvinar–medial frontal gyrus functional connectivity correlated with correct response rates to inconsistent stimulus-result pairs (IGD) left MN–right PCG functional connectivity correlated with inhibition control scores	disrupted thalamocortical communication (imbalance: goal-directed and habitual systems) thalamus (circuit-level) may be a potential IGD biomarker
Chen et al. [90]	34/33 TUD/41 IGD-healthy/33 TUD-healthy	-	resting-state fMRI	↑subcortical–motor network enhanced node strength (IGD and TUD patients) ↑right thalamus–right PCG functional connectivity (IGD and TUD patients) node strength and functional connectivity distinguished disordered participants from HCs	IGD and TUD share common neurological patterns greater connectivity suggests association between rewards and behaviors subcortical–motor network connectivity is a potential treatment target
Dong et al. [91]	149 (23 IGD/23 one-to-one matched non-IGD a year later)	IAT (20)	fMRI (cue-elicited-craving task pre-gaming; gaming; cue-elicited-craving task post-gaming)	↑bilateral lentiform nucleus activation (post-gaming; RGU players developing IGD a year later compared to RGU players who did not develop IGD) lentiform activation correlated positively with self-reported craving (RGU players developing IGD a year later)	post-gaming lentiform activation may be a predictive biomarker of subsequent IGD development
Wang et al. [92]	64/63 RGU	IAT (20)	resting-state fMRI (dynamic causal modeling)	↓mPFC –> PCC effective connectivity ↓left IPL –> mPFC effective connectivity ↓self-connection in PCC and left IPL	dynamic causal modeling distinguishes more precisely IGD decreased mPFC –> PCC excitatory connectivity may be an IGD biomarker
Gong et al. [93]	89 gamers	DSM scale (9)	resting-state fMRI	right VS–dACC functional connectivity correlated with self-control right VS–dACC functional connectivity negatively correlated with number of IGD symptoms self-control influenced IGD symptoms through right VS–dACC functional connectivity right VS connectivity in a reward anticipation limbic pathway contributed to IGD symptoms but not self-control	cingulate–ventral striatal functional connectivity may serve as IGD self-control regulator
Zhang et al. [94]	74/41 (20 IGD with craving behavioral intervention/18 without)	CIAS (26)	resting-state fMRI (second measurement after craving behavioral intervention)	↑VS to left IPL, right IFG, and left MFG functional connectivity (baseline) VS to left IPL, right IFG, and left MFG functional connectivity correlated with IGD severity (baseline) ↓VS–left IPL functional connectivity, greater addiction severity (follow-up IGD–therapy compared to IGD–no therapy)	VS–left IPL functional connectivity may be a biomarker of the efficacy of IGD psychobehavioral intervention noninvasive techniques (transcranial magnetic, direct current stimulation targeting VS–IPL circuitry) may treat IGD
Wang et al. [95]	103/99 RGU	IAT (20)	resting-state fMRI	↓effective connectivity from left parahippocampal gyrus to right MFG ↓effective connectivity from the right parahippocampal gyrus to ACC ↓effective self-connection in right parahippocampal gyrus 82.67% accuracy, 83.50% sensitivity, and 81.82% specificity in distinguishing IGD from RGU (bilateral parahippocampal gyrus, right ACC, MFG) IGD severity correlated positively with ReHo values of discriminating regions	IGD related to weakening of the parahippocampal gyrus and its connection with PFC
Wang et al. [96]	40/19 (23 IGD after 6-week intervention)	CIAS (26)	fMRI (cue reactivity task)	92.37% accuracy, 90.00% sensitivity, and 94.74% specificity in distinguishing IGD from HCs (bilateral MFG, precuneus, posterior lobe of the right cerebellum) MFG, SFG, supramarginal gyrus, anterior/posterior lobes of cerebellum and left PCG predicted craving behavior intervention outcomes	cue reactivity neural correlates identify IGD subjects and predict craving behavior intervention outcomes
Han et al. [97]	20/40/29 IGD+ADHD/	IAT (20)	resting-state fMRI (second measurement after cognitive behavior therapy)	↓cortex–subcortex functional connectivity (baseline, compared to HCs) right insular cortex activity correlated positively with playing time (baseline) left MFG activity correlated negatively with IGD severity (baseline) ↑cortical brain activity within the right MFG (after treatment) ↑cortex and subcortex functional connectivity (groups with good prognosis compared to groups with poor prognosis)	IGD and ADHD have similar functional connectivity at baseline functional connectivity changes after treatment
Wang et al. [101]	123/210	IAT (20)	resting-state fMRI	↓resilience, betweenness centrality, and occurrence in the prefrontal–striatal neural circuit ↓in-degree in DMN circuit	decreased dynamics of the prefrontal–striatal and striatal–DMN neural circuits may be predictive biomarkers of IGD severity (game-craving and game-seeking behaviors)
Zhou [102]	61/61 RGU	IAT (20)	fMRI (cue-elicited craving task)	↓participation coefficients in DMN and frontal-parietal network ↑number of connections between nodes in the ventromedial PFC, ACC, PCC, angular gyrus number of DMN connections positively correlated with addiction severity and craving	IGD related to cognitive control, emotion, and attention deficits during exposition to gaming cues (DMN and frontal-parietal network)
Song et al. [103]	72/41	CIAS (26)	resting-state fMRI	DMN is the most informative network in predicting IGD (classification and regression)	resting-state functional connectivity can predict IGD and its severity (DMN)
Zhou et al. [104]	66/61 RGU	IAT (20)	fMRI (cue-elicited-craving task)	connectome-based predictive modeling (cognitive, attention, and control networks) predicts craving (IGD only) key nodes: SMA, inferior temporal gyrus, insula, OFC	craving network predicts craving scores in IGD
Liu et al. [108]	39/23	CIAS (26)	fMRI (cue reactivity task)	↑VS and DS cue-induced activations VS activity correlated negatively with cue-induced craving (IGD group) duration of IGD correlated positively with activations within the DS (right putamen, pallidum, left caudate) left putamen cue-induced activity correlated negatively with right VS volumes (IGD group)	ventral-to-dorsal striatal cue processing shift
Zheng et al. [109]	82/107/96 TUD	IAT (20)	resting-state fMRI	↑cerebellum functional connectivity (IGD and TUD) ↑dorsal frontostriatal circuit to thalamus effective connectivity (compared to TUD) ↑left striatum–mPFC effective connectivity increased connections in medial prefrontal cortex in both IGD and TUD (higher in TUD)	cerebellum negatively associated with craving reward pathway different in IGD (cortical) and substance addictions (subcortical)
Dong et al. [110]	174/244 RGU	IAT (20)	resting-state fMRI	↓VS–MFG (mostly SMA) functional connectivity ↑DS–MFG functional connectivity left DS–MFG connectivity correlated with IGD severity ↑DS–MFG functional connectivity (longitudinal data)	ventral-to-dorsal striatal shift in development of addiction imbalance within frontostriatal circuits is addiction risk factor
Dong et al. [111]	130/207 RGU	IAT (20)	resting-state fMRI	↓putamen–SFG, MFG, IFG ↓VS–IFG, superior temporal gyrus, MFG negative correlation between frontostriatal functional connectivity and IGD severity and craving	excessive online gaming may stem from poor executive control over game cravings
Hong et al. [112]	12/11	IAT (20)	resting-state fMRI	↓dorsal putamen–posterior insula-parietal operculum functional connectivity more time spent playing online games predicted ↑dorsal putamen–bilateral primary somatosensory cortices functional connectivity in IGD and ↓dorsal putamen–bilateral sensorimotor cortices functional connectivity in HCs	dorsal putamen functional connectivity may be an IGD biomarker
Wang et al. [113]	68/68 RGU	IAT (20)	fMRI (cue-craving task)	correlation in the putamen–MFG–insula pathway in response to game cues in RGUs (absent in IGD) ↑MFG inhibition by the putamen craving scores correlated negatively with putamen–MFG effective connectivity (IGD)	striatum–prefrontal cortex pathway may serve as an IGD risk biomarker
Wang et al. [114]	22/18	IAT (20)	resting-state fMRI (baseline and +6 months later)	↓left DS (putamen)–left insula functional connectivity over time stable right VS (NAcc)–left insula functional connectivity over time left putamen–right NAcc inhibitory effective connectivity in second measurement	longitudinal changes in dynamic coupling between VS and DS future studies on neuromodulation by putamen of the NAcc may bring a crucial prevention/intervention IGD biomarker in high-risk populations
Chen et al. [115]	30/30	CIAS (26)	resting-state fMRI	functional connectivity: ↓left DLPFC–left insula ↓OFC–left insula ↑insula–contralateral insula ↓left DLPFC–left NAcc ↓left DLPFC–right NAcc ↓insula–right NAcc ↑right precuneus–left or right NAcc interhemispheric insula connectivity positively correlated with impulsivity	functional connectivity of insula associated with IGD impulsivity gaming craving through NAcc may not be well-regulated by frontal cortex in IGD
Kim et al. [116]	29/20	IAT (20)	resting-state fMRI fMRI (imageries of gaming and alternative activities)	↑caudate–right parahippocampal gyrus and putamen–right OFC functional connectivity during gaming imagery ↓caudate–right parahippocampal gyrus and putamen–right OFC functional connectivity during alternative ↓NAcc–right precuneus functional connectivity at baseline ↑NAcc–right precuneus functional connectivity post-game unchanged NAcc–right precuneus functional connectivity in post-alternative	IGD network connectivity should be examined during gaming and post-gaming
Han et al. [117]	30/30	CIAS (26)	resting-state fMRI	↓static functional connectivity between right DLPFC and left Rolandic operculum ↑static functional connectivity between right DLPFC and left pars triangularis ↓dynamic functional connectivity between right DLPFC and left insula, right putamen, left precentral gyrus ↑dynamic functional connectivity between right DLPFC and left precuneus dynamic functional connectivity between right DLPFC and left insula correlated negatively with IGD severity	dynamic functional connectivity supplemental to static functional connectivity IGD related to abnormal DLPFC static and dynamic functional connectivity
Dong et al. [118]	154/229 RGU	IAT (20)	resting-state fMRI	↑higher BIS and BAS fun-seeking sensitivity (IGD compared to RGUs) ↓strength in Granger causal connectivity from the right MFG to caudate, from left SFG to centromedial amygdala, and from right superior parietal lobule to left laterobasal amygdala (high BAS/BIS IGD group) ↑strength in Granger causal connectivity from left putamen to right cuneus (high-BAS/BIS RGU group) Granger causal connectivity from centromedial amygdala to MFG mediated directional relationship between behavioral inhibition and Internet addiction	IGD with unbalanced BAS/BIS sensitivity have different connectivity patterns involving amygdala and striatum subdivisions
Ko et al. [119]	30/30	CIAS (26)	resting-state fMRI	↓GM density in bilateral amygdala ↓left amygdala–left DLPFC functional connectivity ↓right amygdala–left DLPFC functional connectivity ↓right amygdala–OFC functional connectivity ↑bilateral amygdala–contralateral insula functional connectivity left amygdala–DLPFC functional connectivity correlated negatively with impulsivity right amygdala–left DLPFC and OFC functional connectivity correlated negatively with impulsivity	reduced GM density in amygdala and amygdala functional connectivity reflect IGD vulnerability (impulsivity)
Lee et al. [120]	18/18	IAT (20)	fMRI (baseline and +12 months later)	↓GM volume in ACC and aMCC (baseline and follow-up) ↓left dorsal putamen–left mPFC functional connectivity (over time, compared to controls) ↑right dorsal putamen–right MOG functional connectivity strength (over time, compared to controls) gaming time per day correlated with changes in dorsal putamen–MOG functional connectivity functional connectivity of DS increased in the mPFC and decreased in the MOG (over time)	weakening of prefrontal control and strengthening of sensorimotor network (changes in DS)
Zhou et al. [121]	41 excessive gaming/78 gaming-naïve	WOW gaming addiction scale	MRI (baseline and +6 months after training)	↓right OFC GM volume (pre-training; compared to gaming-naïve controls) OFC GM volume correlated with addiction severity (pre-training) ↓left OFC GM (gaming training IGD and control groups compared to non-gaming training)	excessive engagement in online gaming marked by OFC structural deficits
Jin et al. [122]	25/21	IAT (20)	resting-state fMRI	↓GM volume in prefrontal cortex (bilateral DLPFC, OFC, ACC, right SMA) ↓bilateral DLPFC, OFC, ACC, SMA–insula, temporal, and occipital cortices functional connectivity ↓bilateral DLPFC, OFC, ACC, SMA–DS, pallidum, thalamus functional connectivity	similar neural mechanisms as in substance dependence (circuit-level) IGD severity modulated by ACC–striatal circuit (VS to DS) OFC circuit (impaired cognitive behavioral performance) associated with IGD severity
Choi et al. [123]	22/25 Internet gaming controls/24 non-gaming controls	IGD scale	MRI	↓GM density in left DLPFC (compared to both control groups) GM density associated with lifetime usage, depressed mood, craving, impulsivity (gaming groups) ↓right NAcc volume (compared to gaming controls only) right NAcc volume associated with lifetime usage and depression	DLPFC serves as a mediator in the association between prolonged gaming and depressed mood neural structures related to reward system associated with IGD
Lee et al. [124]	31/30	IAT (20)	MRI	↓GM volume in ACC and SMA ↓GM volume in left ventrolateral PFC and the left IPL GM volume in ACC and SMA correlated positively with impulsiveness GM volume in left ventrolateral PFC correlated negatively with lifetime gaming	GM abnormalities in areas related to executive control associated with impulsiveness in IGD
Kühn et al. [125]	76 frequent players/78 infrequent players (14-year-olds)	CSV-S (14)	fMRI (monetary incentive delay task)	↑left striatal GM volume (frequent players) left striatal GM volume negatively correlated with deliberation time in Cambridge gambling task ↑striatum activity during feedback of loss (compared to no loss and infrequent players)	IGD related to reward processing (adaptive neural plasticity)
Chen et al. [126]	230 (no groups)	IAT (20)	Structural magnetic resonance	↑GM volumes in the midline components of DMN (right mPFC and precuneus) in more addicted players impaired patterns of structural covariance between DMN-related regions and visuospatial attention and reward craving processing areas	IGD severity related to increased volume of DMN and weakened structural association with visuospatial attention and motivational craving regions
Ma et al. [127]	130/1570/74 TUD	IAT (20)	MRI	overall brain atrophy (TUD); IGD similar trend ↑CT deviations in bilateral fusiform gyrus and lingual gyrus (IGD and TUD) ↓CT deviation in transverse temporal cortex, right parahippocampal gyrus, left lateral occipital cortex (IGD and TUD) ↓CT deviation (IGD) and ↑ CT deviation (TUD) in PCC	IGD and TUD have similar CT developmental pattern CT deviation in PCC differentiates IGD from TUD
Wang et al. [128]	62 (29 males, 33 females)/71 RGU (37 males, 34 females)	IAT (20)	MRI	↑cortical thickness in bilateral rostral MFG, left SFG, left supramarginal gyrus, right superior parietal lobule (males) ↓cortical thickness in bilateral rostral MFG, left SFG, left supramarginal gyrus, right superior parietal lobule (females) ↓cortical thickness in right PCC (males) ↑cortical thickness in right PCC (females) negative correlations among cortical thickness, self-reported cravings, and Internet addiction	males and females may be affected differently by IGD females more vulnerable to IGD (reduced cortical thickness)
Lee et al. [129]	45/35 (males)	IAT (20)	MRI	↓cortical thickness in right rostral ACC, right lateral OFC, left pars orbitalis ↓GM volume in right caudal ACC and left pars orbitalis ↓cortical thickness in right SMA, left frontal eye field, superior parietal lobule, PCC cortical thickness in right lateral OFC correlated negatively with cognitive impulsivity (IGD)	GM alterations reflect risk/reward decision-making and behavioral control problems
Wang et al. [130]	38/66	IAT (20)	MRI	↓cortical thickness in left lateral OFC, IPL, bilateral cuneus, precentral gyrus, right middle temporal gyrus ↓GM volume in left superior temporal gyrus and right supramarginal gyrus	regions associated with cognitive control, decision-making, and reward/loss processing underlie negative outcomes in frequent game-playing
Wang et al. [131]	48/32 (males)	IAT (20)	MRI	↑cortical thickness in bilateral insulae, right inferior temporal gyrus ↓cortical thickness in bilateral banks of the superior temporal sulci, right inferior parietal cortex, right precuneus, right precentral gyrus, and left middle temporal gyrus positive correlation between left insular cortical thickness and IGD severity.	IGD shares structural abnormalities with substance addictions
Tian et al. [132]	-	-	MRI	↑GM volume in bilateral caudate (dorsal anterior, body, tail) and left NAcc ↑WM volume in inferior parietal lobule no WM volume alterations in mesocorticolimbic dopaminergic system GM volume in left lateral orbital gyrus of OFC correlated with game-craving GM volume in the left anterior insula, right NAcc, right caudate, and right OFC correlated with self-control	IGD is associated with GM volume, but not WM volume, in the mesocorticolimbic dopaminergic system
Zhai et al. [133]	16/16	IAT (20)	MRI	↓global efficiency and local efficiency of WM network ↑shortest path length ↓nodal efficiency in frontal cortex, ACC, and pallidum global efficiency of WM network correlated positively with Internet addiction	IGD associated with abnormal topological organization of WM network
Jeong et al. [134]	58/26	IAT (20)	MRI	↑fractional anisotropy values: forceps minor, right anterior thalamic radiation, right corticospinal tract, right inferior longitudinal fasciculus, right cingulum to hippocampus, right inferior fronto-occipital fasciculus ↓radial diffusivity values: forceps minor, right anterior thalamic radiation, inferior fronto-occipital fasciculus playing duration correlated positively with fractional anisotropy values playing duration correlated negatively with radial diffusivity values	increased myelination (higher fractional anisotropy and lower radial diffusivity values) in right-sided frontal fiber tracts may be a biomarker of extended gameplay
Dong et al. [135]	42/44 RGU	IAT (20)	MRI	↑fractional anisotropy values in bilateral anterior thalamic radiation, anterior limb of the internal capsule, bilateral corticospinal tract, bilateral inferior fronto-occipital fasciculus, corpus callosum, bilateral inferior longitudinal fasciculus fractional anisotropy values correlated with Internet addiction severity	IGD associated with increased WM integrity in tracts linking reward circuitry and sensory and motor control systems
Yuan et al. [137]	43/44	IAT (20)	resting-state MRI	↑right caudate and NAcc volume ↓DLPFC–caudate and OFC–NAcc functional connectivity NAcc volume correlated with Internet addiction scores right caudate volume and DLPFC–caudate functional connectivity correlated with impaired cognitive control	similarity between IGD and SUD (striatum volume, frontostriatal circuits functional connectivity)
Seok and Sohn [138]	20/20	IAT (20)	resting-state fMRI	GM volume in the left caudate correlated with IGD severity left caudate–right MFG functional connectivity negatively correlated with IGD severity	IGD associated with neuroanatomical changes in the right middle frontal cortex and the left caudate (reward and cognitive control processes)
Cai et al. [139]	27/30	IAT (20)	resting-state MRI	↑DS (caudate) and VS (NAcc) volume ↑incongruent condition response errors in the Stroop task caudate volume correlated with Stroop task performance NAcc volume correlated with the Internet addiction test score (IGD only)	striatum is implicated in the pathophysiology of IGD
Zeng et. al. [140]	148/169 RGU	IAT (20)	resting-state fMRI	↑OFC–right caudate and right DLPFC–left OFC inhibitory effective connectivity ↑thalamus–left OFC excitatory effective connectivity addiction severity negatively correlated with right OFC–right caudate directional connection	pathway from the right OFC to right caudate (modulation in future IGD interventions)
Wang et al. [141]	133/110	IAT (20)	MRI	20 features from the right caudate nucleus and amygdala, sex, and Internet addiction scores identified IGD patients	radiomics features of subcortical structures and clinical characteristics can serve as an effective tool for distinguishing IGD patients from HCs
Han et al. [142]	59/69	YDQ (8)	MRI	alterations in the bilateral fusiform, left rostral middle frontal gyrus, left cuneus, left pars opercularis, and regions around the right uncinate fasciculus and left internal capsule identified IGD patients no differences in total brain, total GM and WM, subcortical region volumes	IGD associated with brain morphology alterations
Park et al. [143]	102 gaming individuals (51 IGD)/41	-	MRI	configuration of brain structural networks shifted to the direction of random topology (gaming individuals) connection topology of brain structural networks under no attacks comparable to brain structural networks under targeted attacks (gaming individuals vs. non-gaming HCs)	IGD brains may be similar to brains suffering from targeted damage
Wei et al. [144]	49/52	CIAS (26) YDQ (8)	resting-state fMRI	↑synchronizability and modal controllability	brain controllability can be a potential biomarker of IGD (switching to craving states and maintaining them)
Zhang et al. [145]	26/23 not diagnosed excessive players/29	PVP (9) YDQ (8)	resting-state fMRI	↓hubness in the right medial orbital part of the SFG (compared to both other groups) hubness of the right medial orbital part of the SFG correlated with the highest excessive Internet gaming degree during the past year (excessive-not diagnosed only) hubness of the right orbital part of the SFG correlated with IGD treatment outcome (under forced abstinence) ↑impulsivity during the decision-making (IGD) impulsivity-related parameters negatively correlated with the hubness of right orbital part of the SFG (IGD)	impulsivity-related right orbital part of the SFG hubness can be a potential IGD biomarker
Wang et al. [146]	169/147 (36 IGD also 1–6 months later)	IAT (20)	resting-state fMRI	2 subgroups identified craving-related: mostly males; high craving, fMRI cue reactivity, positive results of a craving behavioral therapy ↑insula-PFC functional connectivity mixed psychological: mostly females; high craving, behavioral inhibition and activations scores, non-adaptive emotion regulation strategies, and guessing task fMRI measure ↓striatum–PFC and amygdala–PFC functional connectivity	2 IGD patterns IGD pattern may be sex-related striatum–PFC and amygdala–PFC functional connectivity may underlie negative reinforcement (compulsive gaming; weak game craving regulation)
Zhou et al. [147]	62 (32 males/30 females)	IAT (20)	fMRI (game-related cues)	↓activation in ACC, SFG, and PCC (males compared to females) ↑middle temporal gyrus–PCC–right ACC/parahippocampal gyrus effective connectivity (males compared to females)	gaming cues could more severely disturb males’ inhibition control function over game-elicited cravings
Dong et al. [148]	20/16	IAT (20)	FMRI (risk-taking and risky decision-making tasks)	↓activation of ACC, PCC, middle temporal gyrus (risk-taking task) ↑risk disadvantageous trials ↓activation of IFG and superior temporal gyrus (risky decision-making task) ↓shorter response times (risky decision-making task)	IGD related to impaired executive control
Zeng et al. [149]	78 (39 males)/72 RGU (39 males)	DSM scale (9)	resting-state fMRI	↓modular segregation of the frontal-parietal network (males only) ↑connections between frontal-parietal network and cingulo-opercular network (males only) ↑causal influence from cingulo-opercular network to frontal-parietal network (males only) ↓causal influence from cingulo-opercular network to frontal-parietal network (females only)	poor modular segmentation of the frontal-parietal network and abnormal frontal-parietal/cingulo-opercular network connections underlie male vulnerability to IGD different mechanisms may underlie male and female IGD
Dong et al. [150]	54 (29 male; 25 female)/65 RGU (34 male; 31 female)	IAT (20)	fMRI (game-playing and after forced break)	↓DLPFC–SFG functional connectivity (during playing; males only) ↑striatum–thalamus functional connectivity (during playing; males only) ↓DLPFC–SFG functional connectivity (after forced break) ↓DLPFC–SFG functional connectivity (after forced break; RGU males compared to RGU females) ↑striatum–thalamus functional connectivity (after forced break) striatum–thalamus functional connectivity difference greater in females	executive control areas during playing associated with male IGD functional connectivity during forced breaks relevant to both genders, probably particularly for females
Wang et al. [151]	46 (23 males)/58 RGU (29 males)	IAT (20)	resting-state fMRI	sex-by-group interaction: right PCC, left MOG, right middle temporal gyrus, and right PCG ↑ReHo in left MOG and right middle temporal gyrus (IGD males compared to the same-sex recreational males) ↓ReHo in left MOG and right middle temporal gyrus (IGD females compared to the same-sex recreational females) ↓ReHo in right PCC (only IGD males compared to the same-sex recreational males) ReHo in right PCC correlated negatively with Internet addiction scores (males only)	IGD sex differences are observed in brain regions responsible for executive control, visual and auditory perception
Kim et al. [152]	16/14 AUD/15	IAT (20)	resting-state fMRI	↑ReHo in PCC (IGD and AUD compared to HCs) ↓ReHo right superior temporal gyrus (IGD compared to AUD and HCs) ↓ReHo in ACC (AUD compared to IGD and Hcs) addiction severity correlated positively with ReHo in the medial frontal cortex, precuneus/PCC, left inferior temporal cortex (IGD)	increased ReHo in PCC may be a common feature in IGD and AUD decreased ReHo in superior temporal gyrus may be an IGD biomarker
Sun et al. [153]	30 male and 23 female/30 male and 22 female	CIAS (26) YDQ (8)	resting-state fMRI	↓values in the orbital part of left SFG (amplitude of low-frequency fluctuation; IGD males compared to the male HCs) values in the orbital part of left SFG negatively correlated with impulsiveness ↑orbital part of the left SFG–PCC, right angular gyrus, right DLPFC (functional connectivity in male HCs) orbital left SFG–PCC seed connectivity (IGD males compared to ICD females)	altered amplitude of low-frequency fluctuation values in the orbital part of the left SFG may be a biomarker for the behavioral inhibition function of addicted males
Hou et al. [154]	18/16	IGDS9-SF (9)	F-labeled difluoro-analog of UCB-J (^18^F-SynVesT-1) PET	↓synaptic density in right ACC, bilateral putamen, and right Rolandic operculum synaptic density in bilateral putamen correlated with IGD severity ↑stop-signal reaction times associated with ↓SV2A density in right pregenual ACC and right Rolandic operculum	lower synaptic density contributes to IGD severity and impairments in inhibitory control altered synaptic integrity may be an IGD biomarker
Weinstein [155]	9 abstinent ecstasy users/8	-	single photon emission computed tomography	↓dopamine D2 receptor occupancy of 10.5% in the caudate after playing a motorbike riding game (HCs; follow-up compared to baseline) no change in dopamine D2 receptor occupancy (ex-chronic ecstasy users; follow-up compared to baseline)	decreased sensitivity to natural reward in addicts reduced dopamine response to stimuli (sensitization) in addicts
Koepp et al. [156]	8 volunteers	-	[^11^C]RAC-PET (navigate-a-tank game; looking at empty screen)	↑endogenous dopamine (striatum) during playing ↓binding of raclopride to dopamine recep- tors in the striatum (during game compared to baseline) reduction in binding of raclopride in the striatum correlated with performance level (greatest in VS)	playing makes dopamine released (HCs)
Tian et al. [157]	12/14	IAT (20)	PET + ^11^C-N-methylspiperone	↓glucose metabolism (prefrontal, temporal, and limbic systems) dysregulation of D2 receptors in striatum dysregulation of D2 receptors in striatum correlated with years of overuse D2 receptors level in striatum correlated with decreased glucose metabolism in the OFC	D2 receptor level is associated with glucose metabolism D2/5-HT2A receptor-mediated dysregulation of the OFC may underlie loss of control and compulsive behavior
Kim et al. [158]	23/23	IAT (20)	18F-fluoro-2-deoxyglucose PET	↑impulsivity ↓self-control ↓fluoro-2-deoxyglucose uptake in left medial orbitofrontal gyrus, left MCC, left SFG, right ACC regional cerebral metabolic rate of glucose in right ACC correlated negatively with fulfilled IGD diagnostic criteria	IGD related to glucose metabolism in the prefrontal–cingulate cortices
Klar et al. [159]	25/26	CSAS (18)	MRI	↑levels of glutamate and glutamine (Glx) in striatum	IGD associated with hyperactivation of reward system
McGlade et al. [160]	21 IGD high-risk/19 IGD low risk	IGDS9-SF (9)	proton magnetic resonance MRI	↓N-acetylaspartate levels in right DLPFC history of ADHD predicted lower N-acetylaspartate levels in right DLPFC	ADHD has mediating effect on Internet gaming severity and N-acetylaspartate levels in right DLPFC
Bae et al. [161]	28 IGD+ADHD/27 ADHD/42	IAT (20)	MRI	↓N-acetylaspartate levels in frontal cortex (both ADHD groups) ↑Glx levels (non-IGD ADHD) levels of Glx positively correlated with ADHD and inattention (IGD+ADHD)	IGD with comorbid ADHD associated with hypofrontality
Kim et al. [163]	5 Internet addiction/7	IAT (20)	^11^Craclopride PET	↓dopamine D2 receptor availability in subdivisions of striatum (bilateral dorsal caudate, right putamen)	Internet addiction is associated with dysfunctions in the dopaminergic DS
Yen et al. [166]	69/138	CIAS (26)	genotyping (QIAamp DNA Blood Mini Kit)	catechol-O-methyltransferase (COMT) val158met polymorphism genotype gives 2.09 IGD odds ratio (all participants) IGD–Val/Val genotype relation mediated by impulsivity and fun-seeking	Val/Val genotype is a predictive IGD factor
Ariatama et al. [167]	48 online games players	IGDS9-SF (9)	genotyping (Enzyme-Linked Immunosorbent Assay kit)	IGD–depression one-way relation IGD negatively correlated with dopamine transporter level depression negatively correlated with dopamine transporter level	higher IGD corresponds with lower dopamine transporter level
Paik et al. [168]	63/87	IGD-20 Test (20)	genotyping (TaqMan PCR assay)	*Taq1A ANKK1* and *C957T DRD2* variants not associated with IGD *del*− genotype of the −*141C* associated with excessive gaming and use of gaming to escape from a negative feeling (all males) *del*+ genotype associated with higher novelty seeking (IGD)	*141C* polymorphism may be IGD trait (*del*+ associated with novelty seeking)
Jeong et al. [169]	30/30 (study 1) 31 (alcohol dependence)/29 (study 2)	IAT (20)	genotyping (targeted exome sequencing)	↓frequency of the T allele of rs1044396 in the nicotinic acetylcholine receptor alpha 4 subunit (*CHRNA4*)	T allele of rs1044396 in CHRNA4 is protective against IGD
Kim et al. [170]	30/30	IAT (20) KS-A (15)	genotyping (targeted exome sequencing)	↓rs2229910 of neurotrophic tyrosine kinase receptor, type 3 (NTRK3)	rs2229910 of NTRK3 is protective against IGD.
Yen et al. [171]	69/138	IAT (20)	genotyping (QIAamp DNA Blood Mini Kit)	participants with the TT genotype of rs1137070 had a higher odds ratio for IGD compared to the C carriers expressive hostility behavior and hostility cognition-mediated rs1137070–IGD relation	TT genotype predicts IGD, higher expressive hostility behavior and hostility cognition (lower monoamine oxidase-A activity)
Lee et al. [172]	25/26	K-Scale (15)	genotyping (TaqMan low-density miRNA array)	↓3 miRNA (hsa-miR-200c-3p, hsa-miR-26b-5p, hsa-miR-652-3p) IGD risk ratio: 22.95% (higher ratio with more altered miRNAs) ↑GABRB2 and DPYSL2 expression	hsa-miR-200c-3p, hsa-miR-26b-5p, and hsa-miR-652-3p downregulated
Liang et al. [173]	673 adolescents	IGDS-SF9 (9)	genotyping (saliva DNA extraction kit)	peer victimization-mediated parent–adolescent conflict IGD link OXTR gene rs53576 polymorphism-mediated parent–adolescent conflict IGD link (especially for AA homozygotes carriers; insignificant for G-carriers)	OXTR gene rs53576 polymorphism moderates IGD parent–adolescent conflict relation
Han et al. [174]	79 excessive players/75	IAT (20)	genotyping (blood leukocytes)	↑Taq1A1 allele of the dopamine D2 receptor (DRD2 Taq1A1) and low activity Val158Met in the catecholamine-O-methyltransferase (COMTL) alleles ↑reward dependence presence of the Taq1A1 allele correlated with higher RD scores (excessive players)	IGD related to higher reward-dependency increased prevalence of DRD2 Taq1A1 and COMTL alleles DRD2 Taq1A1 associated with reward dependence
Hong et al. [176]	60 (problematic Internet gameplay +MDD)	IAT (20)	genotyping (blood; G-DEX™ II Genomic DNA Extraction Kit) resting-state fMRI	↑functional connectivity within DMN (mPFC to PCC) (SS-5HTTLPR) ↑functional connectivity within salience network (right supramarginal gyrus to right rostral prefrontal cortex, right anterior insular to right right supramarginal gyrus, ACC to left rostral prefrontal cortex, left anterior insular to right rostral prefrontal cortex) (SS-5HTTLPR) ↑functional connectivity between DMN and salience network (including mPFC to ACC) (SS-5HTTLPR) positive correlation between mPFC and ACC functional connectivity and scores of BIS and Internet addiction scales (SS-5HTTLPR)	DMN–SFG functional connectivity mediates impulsivity SS allele of 5HTTLPR may modulate functional connectivity within DMN and salience network and between the networks serotonergic system may play a role in impulsive game-playing in patients with MDD
Paik et al. [177]	26/25	-	venous blood test (human glutamate ELISA kit, huma dopamine ELISA kit)	↓serum levels of glutamate no difference in dopamine levels serum glutamate and dopamine levels did not correlate with gaming hours and exposure to games (IGD only) serum glutamate levels correlated with the dopamine levels	altered glutamatergic neurotransmission may contribute to IGD glutamate dysfunction may serve as an IGD early-stage biomarker
Cho et al. [178]	24/28	IAT (20)	blood sampling	co-linearly regressed set of plasma metabolites (arabitol, *myo*-inositol, methionine, pyrrole-2-carboxylic acid, aspartic acid) and depression correlated with addiction severity ↓myoinositol, arabitol, glyceric acid ↑mannitol valine–leucine–isoleucine and glycine–serine–threonine pathways were altered in IGD; alanine–aspartate–glutamate and arginine–proline metabolisms were differentially regulated in ADHD	ADHD includes depression as a clinical parameter, IGD anxiety myoinositol and arabitol most linked to IGD
Choi et al. [179]	33/40	IGDQ (9)	blood sampling	↑orexin A ↑melatonin (*p* = 0.055) BDNF expression negatively correlated with increased gaming time (IGD only) soluble intracellular adhesion molecule-1 (sICAM-1) positively correlated with increased gaming time (IGD only)	BDNF—Internet gaming time negative correlation and increased orexin-A may be IGD biomarkers
Jang et al. [180]	34/30 AUD/35	IAT (20)	blood sampling	↑serum levels of kynurenine (IGD and AUD) ↓kynurenine acid/kynurenine ratio (IGD and AUD) kynurenine levels and kynurenine acid/kynurenine ratios intermediate between AUD and HCs	IGD associated with suggested stress-related psychoimmunological changes
Lee et al. [181]	IGD and HC serum samples	-	blood sampling	↑5-HT levels	biosensor for 5-HT can be used as potential IGD diagnostic method
Lee et al. [182]	61/28	IAT (20)	blood sampling (liquid-chromatography Orbitrap mass spectrometry)	19 lipids dysregulated (compared to controls) 2 lysophosphatidylcholines (16:0 and 18:0) showed highest linearity with IGD score	2 lysophosphatidylcholines (16:0 and 18:0) may be IGD biomarker
Kornhuber et al. [184]	27 risk/addicted/27 unproblematic	CSAS II (14)	2D:4D finger ratio measurement	↓mean 2D:4D values	problematic video gaming related to prenatal androgen exposure and a hyper-male brain organization
Müller et al. [185]	217 participants	S-IAT (12)	2D:4D finger ratio measurement	lower IGD associated with female hands (lower prenatal testosterone, higher 2D:4D ratio) negative association between loss of control of generalized Internet use disorder and 2D:4D ratio (males)	2D:4D marker is an interesting marker for Internet addiction
Chi and Hsiao [187]	7 high GD-risk/17 low GD-risk	CIAS (26) IGDQ (9)	PRV; arterial blood pressure (gameplay videos with negative or positive emotional stimuli)	↑normalized very high-frequency PRV and instantaneous respiratory frequency (while watching negative or positive gameplay stimuli, compared to baseline) ↓normalized low-frequency PRV and low-frequency/high-frequency PRV ratio (while watching negative or positive gameplay stimuli, compared to baseline) ↑normalized very high-frequency PRV (while watching negative gameplay stimuli) ↓normalized low-frequency PRV and low-frequency/high-frequency PRV ratio (while watching negative gameplay stimuli)	indexes of instantaneous PRV and instantaneous respiratory frequency can be used to detect GD
Long et al. [188]	20 high-risk gamers/22 low-risk gamers	CIAS-R (26)	HRV (while playing a game)	↑low-frequency power/high-frequency power ratio (all game phases) Internet addiction score predicted low-frequency power/high-frequency power ratio (all game phases) number of times being slain in all game phases predicted HRV indicators of sympathetic activity number of times being slain in all game phases and Internet addiction score predicted HRV indicators of parasympathetic activity	IGD risk associated with dysregulated autonomic balance during gameplay
Long et al. [189]	42 habitual players	CIAS-R (26)	HRV (while playing a game)	Internet addiction score predicted HRV indices representing autonomic balance HRV indicators of sympathetic activity correlated with game performance (whole game) HRV indicators of parasympathetic/vagal activity correlated with game performance, ranking score, and Internet addiction score ↑sympathetic activation in the early and late game phases (IGD high risk)	IGD-related autonomic dysregulation may stem from complex interactions between personal attributes and game mechanics
Hong et al. [190]	21/27	IAT (20)	HRV (resting state and while playing a game)	↓high-frequency HRV (compared to baseline, particularly during periods of high attention and last 5 min) increased high-frequency HRV predicted by IGD symptom score	diminished executive control involved in IGD HRV response to gaming situations related to addictive gaming
Hong et al. [191]	70 (diagnosed 15 normal, 30 mild, 23 moderate, 2 severe)	IAT (20)	HRV (while playing a game)	high-frequency HRV predicts IGD severity classification (deep learning model input) low-frequency HRV and high-frequency HRV identified as regions of interest in the deep learning model (severe IGD group)	parasympathetic tone reflects executive control dysfunction (losing control)
Lee et al. [192]	23/18	IAT (20) K-Scale (15)	HRV (while playing a game) MRI	↓high-frequency HRV during game-playing no differences in DLPFC and ventrolateral PFC GM volume high-frequency HRV decrease correlated positively with IGD severity and prefrontal GM volume (IGD only) high-frequency HRV decrease negatively correlated with prefrontal GM volume (IGD only)	IGD associated with difficulties in executive control over gaming
Chang et al. [193]	22 excessive online gaming/22	IAT (20)	ECG (action video game play)	↓cardiorespiratory coupling (i.e., heart rate and respiration) severity of cardiorespiratory coupling associated with problematic Internet use	impaired cardiorespiratory coupling in problematic Internet use excessive gaming type
Kim et al. [194]	38/30	Online Gaming Addiction Scale for Adolescents (20)	HRV (resting state)	↑type D personality most had type D personality (46 of 68) type D personality negatively correlated with logarithmic value of total power and low frequency among the HRV parameters	excessive Internet gaming related to alterations in autonomic functions and D personality
Kim et al. [195]	57 mild to severe IGD	IAT (20)	photoplethysmogram, galvanic skin response, electrooculogram (while watching game clips or natural scenery)	in individuals with higher self-reported craving scores: ↓standard deviation of the heart rate ↓number of eye blinks ↓number of saccadic eye movements ↑mean respiratory rate participants with higher Internet addiction had higher craving scores classification of craving for gaming with an average accuracy of 87.04%	electrooculogram can give markers of game craving
Park et al. [196]	53/61/49 AUD	IAT (20)	resting-state HRV	↓standard deviation of the normal-to-normal beat interval (IGD and AUD compared to HCs)	IGD and AUD individuals have lower HRV compared to HCs IGD and AUD individuals have higher vulnerability to stress compared to HCs
Ono et al. [197]	14/21	IAT (20)	ECG (24 h and during IGT and favorite game)	depressive symptoms, anxiety, impulsivity, autistic tendencies, and sleep problems in excessive Internet user group (compared to non-excessive group) ↓high-frequency response during IGT in high-school students compared to baseline	average high frequency was higher in the excessive Internet gaming group, which was more likely to be depressed and to have pre-Internet tendencies
Wang et al. [199]	57 high-risk IGD/52 low-risk IGD	CIAS (26) IGDQ (9)	eye tracking-based anti-saccade task	↓correct rate of eye tracking anti-saccade task ↑Barratt impulsiveness scale score ↓WLEIS combination of WLEIS score and correct rate of anti-saccade task could discriminate high-risk players from low-risk ones	combination of abnormal emotion regulation and response inhibition may be a biomarker of IGD high-risk playing
Kim et al. [200]	23/27	IAT (20)	eye tracking-based anti-saccade task (game-related, neutral, scrambled images)	↑error rates (game-related images vs. neutral or scrambled ones; IGD group only)	IGD associated with deficits in goal-directed behavior or inhibitory control attentional bias toward game-related stimuli may be an IGD biomarker
Jeong et al. [201]	28/24	IAT (20)	MK-SVM (EEG, PET, psychometric variables)	86.5% accuracy, 89.3% sensitivity, 83.3% specificity of IGD prediction	MK-SVM gives better IGD prediction than other conventional machine learning methods
Ha et al. [202]	18/15 rare game players/18 non-IGD regular game players	IAT (20)	EEG, eye tracking, HRV	90% accuracy in IGD prediction (vertical saccadic movement, standard deviation of normal-to-normal intervals, pNN50, prefrontal theta power, prefrontal alpha power, prefrontal delta/gamma ratio, prefrontal delta/beta ratio, prefrontal theta/beta ratio, prefrontal alpha/beta ratio, frontal alpha/beta ratio, parietal delta/gamma ratio, occipital delta/gamma ratio, and occipital theta/gamma ratio)	13 physiological features can predict IGD

## Data Availability

Data sharing is not applicable. No new data were created or analyzed in this study. Data sharing is not applicable to this article.
